# Optimization of wear parameters for ECAP-processed ZK30 alloy using response surface and machine learning approaches: a comparative study

**DOI:** 10.1038/s41598-024-59880-0

**Published:** 2024-04-22

**Authors:** Mahmoud Shaban, Fahad Nasser Alsunaydih, Hanan Kouta, Samar El-Sanabary, Abdulrahman Alrumayh, Abdulrahman I. Alateyah, Majed O. Alawad, Waleed H. El-Garaihy, Yasmine El-Taybany

**Affiliations:** 1 Department of Electrical Engineering, College of Engineering, Qassim University, 56452 Unaizah, Saudi Arabia; 2 Department of Electrical Engineering, Faculty of Engineering, Aswan University, Aswan, 81542 Egypt; 3 Department of Production Engineering and Mechanical Design, Port Said University, Port Fouad, 42526 Egypt; 4 Department of Mechanical Engineering, College of Engineering, Qassim University, 56452 Unaizah, Saudi Arabia; 5 Center of Excellence for Nanomaterials for Clean Energy Applications, King Abdulaziz City for Science and Technology (KACST), 12354 Riyadh, Saudi Arabia; 6 Mechanical Engineering Department, Faculty of Engineering, Suez Canal University, Ismailia, 41522 Egypt

**Keywords:** Equal channel angular pressing, ZK30 alloy, Wear performance, Response surface methodology, Machine learning, Engineering, Materials science, Mathematics and computing

## Abstract

The present research applies different statistical analysis and machine learning (ML) approaches to predict and optimize the processing parameters on the wear behavior of ZK30 alloy processed through equal channel angular pressing (ECAP) technique. Firstly, The ECAPed ZK30 billets have been examined at as-annealed (AA), 1-pass, and 4-passes of route Bc (4Bc). Then, the wear output responses in terms of volume loss (VL) and coefficient of friction (COF) have been experimentally investigated by varying load pressure (P) and speed (V) using design of experiments (DOE). In the second step, statistical analysis of variance (ANOVA), 3D response surface plots, and ML have been employed to predict the output responses. Subsequently, genetic algorithm (GA), hybrid DOE–GA, and multi-objective genetic algorithm techniques have been used to optimize the input variables. The experimental results of ECAP process reveal a significant reduction in the average grain size by 92.7% as it processed through 4Bc compared to AA counterpart. Furthermore, 4Bc exhibited a significant improvement in the VL by 99.8% compared to AA counterpart. Both regression and ML prediction models establish a significant correlation between the projected and the actual data, indicating that the experimental and predicted values agreed exceptionally well. The minimal VL at different ECAP passes was obtained at the highest condition of the wear test. Also, the minimal COF for all ECAP passes was obtained at maximum wear load. However, the optimal speed in the wear process decreased with the number of billets passes for minimum COF. The validation of predicted ML models and VL regression under different wear conditions have an accuracy range of 70–99.7%, respectively.

## Introduction

Magnesium (Mg) has demonstrated an impressive role in a wide range of engineering sectors due to its unique properties. Mg is the lightest weight amongst other metals with a density of only 2/3 of aluminum; therefore, it has countless applications in cases where weight reduction is essential, i.e., automotive, aerospace, and structural industries^1–3^. In addition, Mg has a high strength-to-weight ratio, high damping capacity, and good machinability. Furthermore, its remarkable biological and mechanical properties make Mg a promising biodegradable material that has increasingly emerged in recent biomedical applications, including orthopedic implants and cardiovascular stents^4,5^. Mg exhibits mechanical properties similar to human bone, such as density and elastic modulus, and fully degrades in the human body. So, no additional surgery is needed for implant removal after the healing of bone tissue. Moreover, Mg shows extreme compatibility with bone cells and doesn’t pose a toxicity risk^6,7^.

Despite all the aforementioned merits, the high corrosion rate remains Mg’s major inherent limitation, which is a significant barrier to further biological applications. For instance, Mg corrodes rapidly in the chloride medium inside the human body, leading to fast degradation, mechanical support distortion, and, consequently, failure of the Mg implant before the healing process. Additionally, the corrosion process releases some toxic elements and produces hydrogen gas bubbles that accumulate in the body, causing damage to the implant sites^8,9^. Hence, improving Mg’s mechanical and biological performance is a challenging endeavor that has gained a lot of interest from the scientific and medical communities. From this perspective, many attempts have been made to find effective methods for producing biomaterials with the required properties, high biological safety, and reliable performance to develop new biomedical applications. According to the published articles, enhancing the mechanical and corrosion behavior of Mg used for biomedical applications, where friction and wear are involved, can be accomplished by applying either metallurgical or surface modification techniques^10–12^.

Surface microstructural modification is achieved through mechanical processing, specifically utilizing severe plastic deformation (SPD) techniques such as high-pressure torsion (HPT), multi-directional forging (MDF), and equal channel angular pressing (ECAP)^13^. Notably, ECAP surpasses other SPD methods in effectiveness as it produces a homogeneous ultrafine-grained (UFG) structures. This enhancement in microstructure contributes to improved mechanical performance, wear and corrosion resistance without compromising biological response^14,15^. Alateyah et al. demonstrated that processing pure Mg through 2-passes of ECAP led to significant ultrafine structure^1^. Sahoo et al.^16^ demonstrated a 70% grain refinement in Mg-RZ5 alloy and a 12% increase in strength and 16% improvement in hardness through four-passes of hot ECAP. El-Garaihy et al. experimentally investigated the effect of different ECAP parameters on the performance of ZK30 Mg alloy^7^. Additionally, prediction models using a machine learning approach were created to estimate the ECAP parameters, validating the experimental optimum results^17^. The impact of varying ECAP processing parameters on the mechanical and electrical behaviors of pure copper (Cu) was studied numerically, experimentally, and statistically. Using ECAP dies with angles of 90° and 120°, processing routes (A, Bc, and C), at room temperature, 100 °C, and 200 °C up to 6-passes, the results demonstrated that the 6-Bc route using ~ 110°-die angle at ~ 190 °C was the optimum condition significantly improving grain size, hardness, and ductility^18,19^. In contrast, Vaughan et al.^20^ reported that ECAP induced grain refinement in Mg-ZKQX6000 alloy but deteriorated its corrosion resistance. In addition, the significance of the ultrafine structure produced through ECAP on wear characteristics is rarely discussed, highlighting the need to emphasize the importance of adapting processing parameters.

Prior studies employed various statistical techniques such as response surface methodology (RSM), genetic algorithms (GA), hybrid design of experiments and GA, and multi-objective genetic algorithms to optimize ECAP analysis. By looking at thirty-one tests created by RSM to look into the ECAP process parameters, Daryadel^21^ verified the finite element simulation of the ECAP process of AA7075 with copper casing. The ECAP angle was the most effective ECAP input parameter since, according to the ANOVA analysis, it was anticipated to have the most effect on the response. Alateyah et al.^22^ optimized the ECAP parameters of pure Mg using RSM, ANOVA, GA, and RSM-GA. They reported that the most significant parameters in grain refining and Vicker’s microhardness values were obtained via ECAP processing using a die with ɸ = 90° through 4-passes of route Bc.

Recently, machine learning (ML) is a form of artificial intelligence focused on creating algorithms that enable computers to learn and make predictions without explicit programming^23^. ML involves constructing systems capable of analyzing and spotting patterns in data to make informed decisions. ML algorithms learn from historical data, using statistical approaches to recognize patterns, connections, and trends^24^. There are various ML approaches, such as supervised, unsupervised, semi-supervised, and reinforcement learning. Supervised learning, the most common, analyzes training data to identify trends and make forecasts based on historical data^25^. Unsupervised learning involves preparing data, model setup, feature extraction, algorithm selection, training, confirmation, and testing, often working best in combination with supervised learning. It uses the dataset for training to identify effective model parameters, revealing previously unrecognized relationships.

In an ideal world, data from neither the testing nor the training stages would be used to adjust the hyper parameters^26^. Overfitting is a prevalent problem during model training, in which the model matches the dataset excessively without considering the regularization method^27^. In such cases, the trained model seldom performs well during testing validation. When dealing with a small set of data, like the one employed in this study, cross-validation (CV) is utilized to address over fitting issues. The k-fold CV technique divides the training data into several independent subsets, or “folds”. Each fold is used to train the model, while the remaining data is used to evaluate its performance. This technique is looped k times, and the model’s success is determined by averaging the data values across iterations. Although computationally costly, this strategy aids in data preservation, especially when working with small-size datasets^28^. From this point of view, controlling the ECAP processing parameters is crucial as they directly influence the microstructural, mechanical, and wear behavior. From the above literature, in the current work, the main aim is to predict and optimize the ECAP parameters on the wear behavior of ZK30 alloy using statistical analysis and machine learning approaches.

## Experimental specifics and methodology

### Materials and experimental procedures

**Figure 1:**
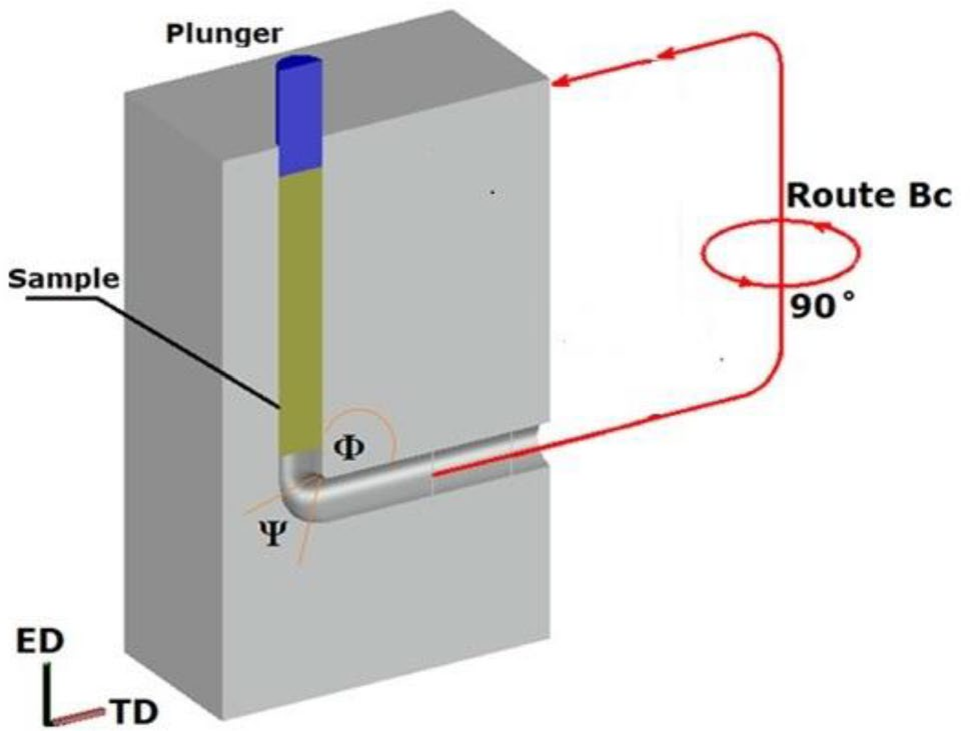
Schematic of ECAP process.

In this study, Mg-3Zn-0.6Zr-0.4Mn, wt% (ZK30) alloy billets were machined with 20 mm diameter and 60 mm length. The ZK30 billets were annealed before ECAP processing for 16 h at 430 °C, followed by furnace cooling. ECAP processing was conducted using a die with an internal channel angle of Φ = 90° and curvature angle of Ψ = 20° (Fig. 1). To regulate temperature, the dies were wrapped with a heating element and insulated using a ceramic fiber layer. Temperature measurements were conducted with K-type thermocouples. To ensure uniform temperature distribution during extrusion, monitoring was performed before and throughout the process, revealing a temperature variation of only 3 °C along the inlet channel, and ECAPed at 250 °C. Prior to extrusion, the samples remained in the die for 15 min to attain a steady-state processing temperature. A universal testing machine (Shimadzu 100kNXplus) applied the pressing load and controlled the speed, with a constant ram speed set to 1 mm/s for all experiments. The ZK30 billets processed through a single pass (1-P) and four passes of route Bc (4Bc), with the sample rotated 90° between subsequent passes.

Preparing samples for metallographic analysis involved standard mechanical grinding and polishing procedures for both the as-annealed (AA) and ECAP-processed samples. ZK30 billets were sectioned and mounted in conductive epoxy. ZK30 samples were grinded incrementally using silicon-carbide sandpaper (600/800/1000/1200 grit), samples were washed with water and dried using alcohol before switching to higher grit sandpaper. Then the samples were polished using diamond suspensions of particle sizes 6 μm followed by 1 μm. To that end, a final polishing step was conducted; a 0.05-micron colloidal silica formula was used to provide the final polish. Between polishing rounds, the specimen was ultrasonically cleaned in ethanol for 10 min. The ZK30 samples were etched using a solution comprising 6 g picric acid, 5 mL acetic acid, 100 mL ethanol, and 10 mL deionized water. For electron backscattered diffraction (EBSD), the longitudinal plane was interfaced with a scanning electron microscope (SEM) to acquire grain size and grain orientation distribution maps for all ZK30 samples. These data were subsequently processed through the HKL Flamenco Channel 5 software program (Hitachi, Ltd., Tokyo, Japan). To ensure robust data acquisition for meaningful statistical analysis, the SEM operated at 15 kV and 1.5 nA, with a 100 nm step size from the extrusion direction (ED) surface during EBSD. Furthermore, X-ray diffraction (XRD; 6100 Shimadzu) equipped with a CuKa radiation source having a wavelength (k) of 1.5418 was used to analyze the phases structure from 20° to 80°.

The wear behavior of ECAPed ZK30 billets was investigated using a ball-on-flat apparatus (Tribolab, Bruker’s universal mechanical tester, USA). The wear behavior of ZK30 alloy was studied under three different applied loads (1, 3, and 5 N) based on previous studies and according to the material response. Due to the sample diameter of 20 mm which resulted in adapting the stroke for the ball as max 10 mm, since the surface have several tests. The issue comes from the ability of the rotary drive of the Triolab machine since the stroke is short so the speed reaches the maximum rotation speed that transfer the movement of the sample to be reciprocating movement. To that end, three different speeds (64.5, 125, and 250 mm/s) were selected to examine the effect of wear speed on each coefficient of friction and volume loss. Furthermore, the ZK30 samples were tested for 110, 210 and 410 s. As the wear test parameters vary between the force and the speed, the time was selected to maintain the same distance was done by all the conditions. So in in case of speed 64.5 mm/s the time is 410 s, while in case of speed 125 mm/s the time is 210, and for the speed of 250 mm/s the time is 110 to maintain the same wear distance for all wear speeds. All ZK30 samples were ground and polished to a mirror-like finish before performing wear tests. The volume loss and coefficient of friction were measured and analyzed, and the average values were calculated for all the wear parameters of ECAPed ZK30 samples.

### Statistical analysis of variance (ANOVA)

Examination of variation in the current investigation, ANOVA was employed to analyze the practical data and determine which variables had the most important impacts of the input parameters (P and V) on the outcomes of the output responses (VL and COF). The Design Expert software has been used during the statistical analysis. An overview of the ANOVA results is provided in Table 1. At a 95% confidence level, the adjusted R^2^, expected R^2^, *p* value, adequate precision, and F-value are reported. All the responses had *p* values that were less than 0.05 and F-values that were larger than 4, suggesting that the predicted models were adequate and that the independent parameters, individual model coefficients, and interaction terms significantly influenced the responses received. For AA, one and four passes, velocity significantly affected VL and COF followed by pressure. To evaluate the validity of the model, the signal-to-noise ratio (S/N) was estimated with adequate precision. The S/N ratio should be greater than four. Because the appropriate precision of the obtained responses was greater than four, suggesting sufficient signal, the model can be utilized for negotiating the design space.

**Table 1:** Wear responses analyzed using an ANOVA.

No. of Pass	Response	F-value (F > 4)	*p* value (*p* < 0.05)	Adeq Precision (ratio > 4)	R^2^	${\text{R}}_{{{\text{adj}}}}^{2}$	${\text{R}}_{{{\text{pred}}}}^{2}$
AA	VL	111.06	< 0.0001	30.2695	0.9788	0.9700	0.9567
	COF	14026.08	< 0.0001	333.9971	0.9999	0.9999	0.9997
1P	VL	577.91	< 0.0001	82.8475	0.9975	0.9958	0.9934
	COF	1792.21	< 0.0001	118.5669	0.9987	0.9981	0.9972
4P	VL	2459.40	< 0.0001	130.1004	0.9994	0.9994	0.9984
	COF	176.72	< 0.0001	40.8852	0.9866	0.9810	0.9717

### Machine learning (ML) approach

In order to predict the ECAP properties of ZK30 alloys, a precise predictive machine learning model was created. The basic methodologies for constructing these models were linear regression (LR), random forest (RF), Gaussian process regression (GPR), support vector machine for regression (SVR), and gradient boosting (GBoost) algorithms^29–33^. The combination of these ML models holds excellent potential for accurately predicting the ECAP parameters, showing significant promise. They use algorithms to discover traits, correlations, and patterns in the data being researched. Some of these techniques are discussed in the following context:

#### Linear regression (LR)

Linear regression is a simple ML technique that seeks to predict the connection between a dependent variable and one or more independent variables by fitting a linear equation to the observed data. The aim is to select the best-fitting line that minimizes the difference between anticipated and actual dependent variable values. The linear regression equation is given by:

1 $$y{ } = { }\mathop \sum \limits_{i = 1}^{n} b_{i} x_{i} { } + { }b_{0}$$

where y is the dependent variable, x_1_, x_2_,…, x_n_ are the independent variables, b_0_ is the intercept, and b_1_, b_2_,…, b_n_ are coefficients that represent the relationship between the independent variables and the dependent variable. The model is trained by predicting the best coefficient values using a method known as ordinary least squares, which minimizes the sum of squared discrepancies between predicted and actual values. Once trained, the model may be used to make predictions by changing the independent variables' values. In some cases, the model is overfitted due to the simplicity of the data pattern. In order to provide a regulated model, a method called regularized linear regression (RLR) is commonly used^27^. It seeks a linear connection between the input and goal variables while minimizing the sum of squared errors and a penalty term. By adding a regularization parameter multiplied by the L1 norm (Lasso regularization) or L2 norm (Ridge regularization) of the regression coefficients, the penalty term helps to regulate the model’s complexity. In Ridge regularization, the loss function (L) can be computed using:

2 $$L{ } = { }\left( {y{ } - { }Xb} \right){^{\prime}}\left( {y{ } - { }Xb} \right){ } + { }\lambda { * }b{^{\prime}}b$$

where *y* is the vector of observed dependent variable values, *X* is the matrix of independent variables, *b* is the vector of coefficients, and λ is the regularization parameter, (*y* − *Xb*)' is the transposition of the difference between the observed and predicted values, and *b'b* is the coefficient vector of the squared L2 norm.

#### Support vector machines (SVM)

SVM is a supervised learning method used for classification and regression problems. SVM seeks an optimum hyperplane that best divides data points from distinct classes or predicts the target variable. In the case of binary classification, SVM selects the hyperplane with the most significant margin between the two classes' closest data points^34^. The linear SVM characteristic equations are listed as follows:

3 $$f\left( x \right) = wx + b$$

where *w* represents the weight vector, *x* is the input vector, and *b* is the bias term. The optimized values of *w* and *b* can be acquired by minimizing the following term:

4 $$\min \;\frac{1}{2}\left\| W \right\|^{2} + C\mathop \sum \limits_{i = 1}^{N} \left( {\xi_{i} + \xi_{i}^{*} } \right)$$

with the following constraints:

5 $$\left\{ {\begin{array}{*{20}l} {y_{i} - wx_{i} - b \le \varepsilon + \xi_{i} } \hfill \\ {wx_{i} + b - y_{i} \le \varepsilon + \xi_{i}^{*} } \hfill \\ {\xi_{i} , \xi_{i}^{*} \ge 0} \hfill \\ \end{array} } \right.$$

where *ε* indicates an error tolerance, and *C* is a compromise between the empirical error and the general term.

#### Gradient boosting (GBoost)

GBoost is an ensemble approach for creating a robust predictive model by combining numerous weak predictive models (usually decision trees). It constructs the model iteratively, with each successive model focusing on addressing the errors created by earlier models. The final prediction is derived by adding the weak models' predictions and weighting them with a learning rate. The Gboost approach optimizes a loss function (e.g., mean squared error) by repeatedly fitting weak models to the loss function’s negative gradient^33^. The goal of GBoost is to create an approximation of the underlying function F*(*x*) that translates instances *x* to their associated output values *y*, denoted as *F*(*x*). This goal is accomplished by utilizing a training dataset to minimize the expected value of a specified loss function. These fundamental functions can be represented by models such as decision trees as follows:

6 $$y_{i + 1} = y_{i} + \alpha {*}F_{i} \left( x \right)$$

where *y*_*i*_ represents the prediction at iteration *i*, *α* is the learning rate, which is a hyperparameter controlling the contribution of each weak model, and *F*_*i*_(*x*) is the weak model’s prediction at iteration *i*.

#### Random forest (RF)

RF is an ensemble learning approach that builds many decision trees and combines their forecasts to generate a final prediction. Each decision tree is constructed using a random selection of training data characteristics and samples. The final prediction is generated by aggregating all individual tree forecasts (e.g., a majority vote for classification or average for regression)^31^. The random forest prediction equation is:

7 $${\text{y}} = mode\left( {y1,y2,...,yn} \right)$$

where *y*_*i*_ represents the prediction of each individual tree, and mode returns the most frequent prediction for classification or the average for regression.

#### Gaussian process regression (GPR)

GPR is a non-parametric probabilistic regression approach that models the connection between input and target variables. It considers predictions as a Gaussian process, with a mean and a covariance function (kernel) defining the range of probable functions. GPR generates a posterior distribution over the predicted functions, allowing for the assessment of uncertainty^32^. A GP is often defined by its mean function, *m*(*x*), and covariance function (also known as the kernel function), *k*(*x*, *x'*), where *x* and *x'* are two occurrences inside the input features matrix *x*. As a result, the expected y* values may be described as a Gaussian process function as follows:

8 $$y^{*} { }\sim { }GP\left( {m\left( x \right),k\left( {x,x{^{\prime}}} \right)} \right)$$

Overfitting and underfitting are common challenges encountered in machine learning modeling. These problems can be treated as follows: Feature Selection which includes selection the most relevant characteristics to reduce model complexity. This helps to keep the model from fitting noise in the data. Adding a regularization term, such as L1 (Lasso) or L2 (Ridge) regularization, to the loss function. Regularization increases the model’s complexity, deterring overfitting. Cross-validation that evaluate model performance on different data subsets. If the model’s performance differs dramatically among folds, this might imply overfitting. Early Stopping which refers to evaluate the model’s performance on a validation set while training. Stop the training process when the validation error begins to rise, indicating that the model is overfitting the training data. To overcome underfitting, however, feature extraction or modification may be thought of as representing complex interactions between input and output variables. Model complexity can be raised to reflect underlying data patterns by including higher-order or interaction variables in the model. Ensemble methods, such as bagging, boosting, or stacking, can be used to combine different models to improve prediction accuracy. Data augmentation strategies may be used to enhance the quantity of the training data, exposing the model to a wider range of patterns while decreasing underfitting. To address these issues in our model, we used cross-validation and model complexity techniques.

## Results and discussions

### Experimental results

#### Microstructure evolution

**Figure 2:**
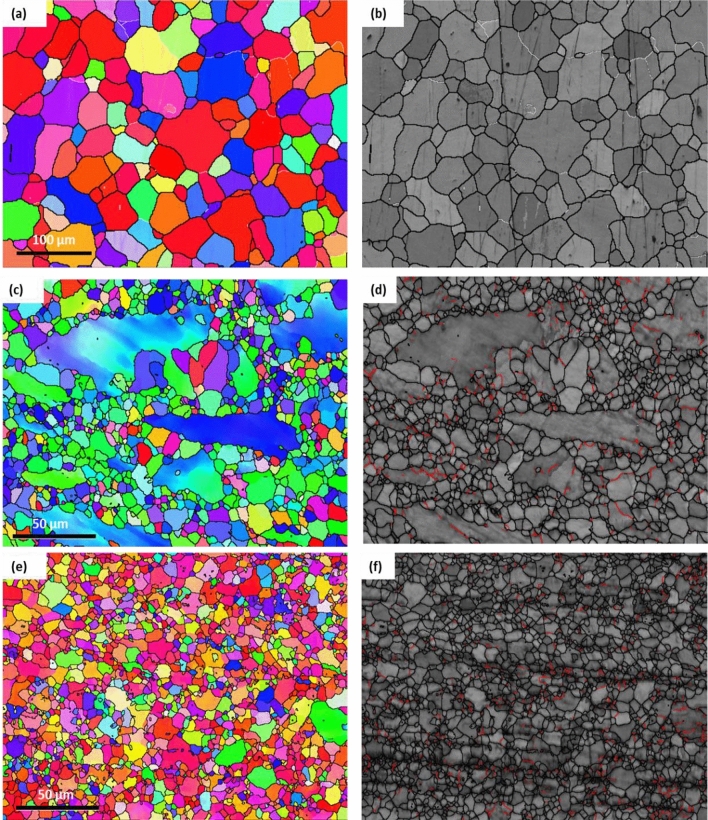
IPF coloring maps and their corresponding BC maps, superimposed for the ZK30 billets in its AA condition (**a**,**b**), and ECAP processed through (**c**,**d**) 1P, (**e**,**f**) 4Bc (with HAGBs in black lines and LAGBs in white lines (AA) and red lines (1P, 4Bc).

**Figure 3:**
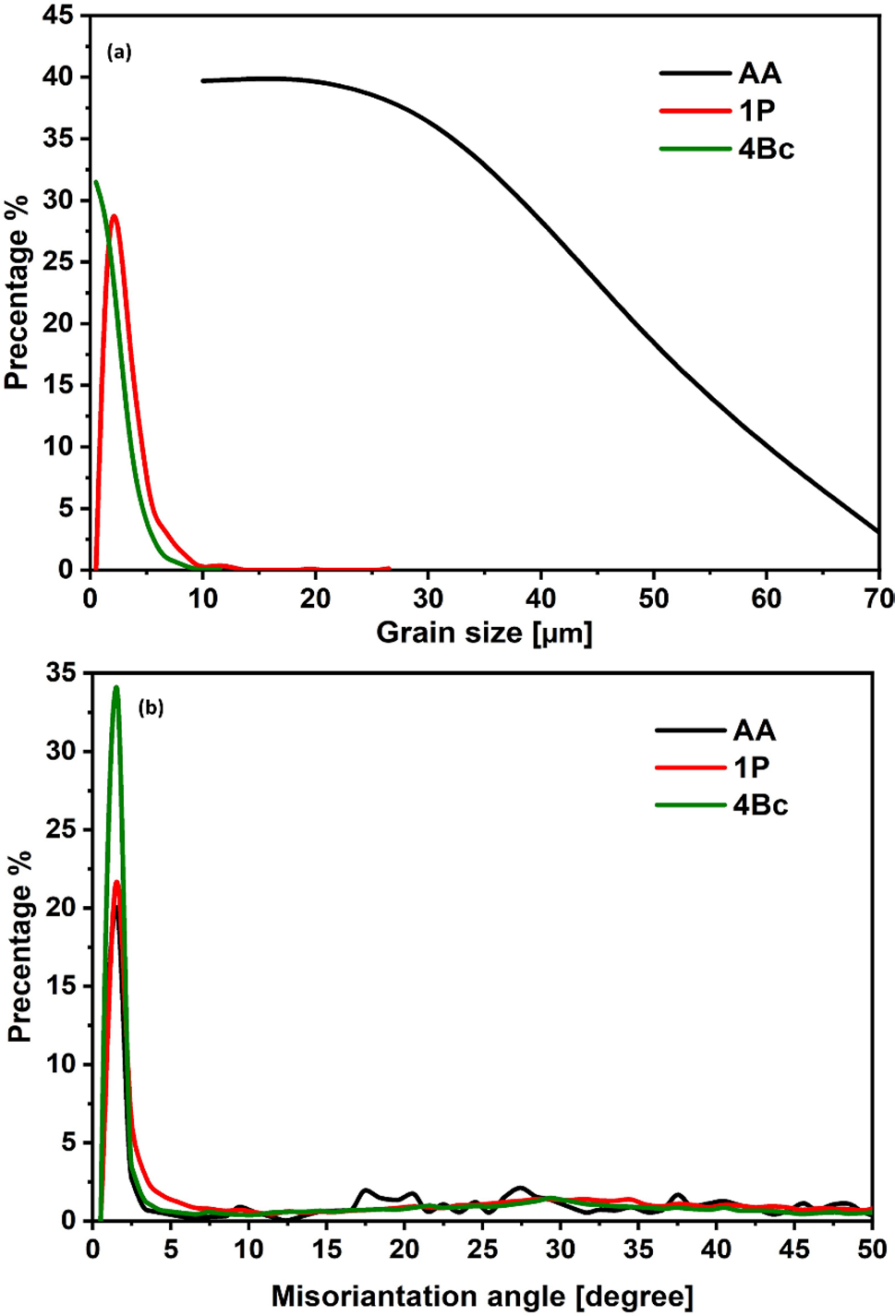
Relative frequency of (**a**) grain size and (**b**) misorientation angle of all ZK30 samples.

The ZK30’s AA and ECAPed conditions of the inverse pole figures (IPF) coloring patterns and associated band contrast maps (BC) are shown in Fig. 2. High-angle grain boundaries (HAGBs) were colored black, while Low-angle grain boundaries (LAGBs) were colored white for AA condition, and it was colored red for 1P and Bc, as shown in Fig. 2. The grain size distribution and misorientation angle distribution of the AA and ECAPed ZK30 samples is shown in Fig. 3. From Fig. 2a, it was clear that the AA condition revealed a bimodal structure where almost equiaxed refined grains coexist with coarse grains and the grain size was ranged between 3.4 up to 76.7 µm (Fig. 3a) with an average grain size of 26.69 µm. On the other hand, low fraction of LAGBs as depicted in Fig. 3b. Accordingly, the GB map (Fig. 2b) showed minimal LAGBs due to the recrystallization process resulting from the annealing process. ECAP processing through 1P exhibited an elongated grain alongside refined grains and the grain size was ranged between 1.13 and 38.1 µm with an average grain size of 3.24 µm which indicated that 1P resulted in a partial recrystallization, as shown in Fig. 2c,d. As indicated in Fig. 2b 1P processing experienced a refinement in the average grain size of 87.8% as compared with the AA condition. In addition, from Fig. 2b it was clear that ECAP processing via 1P resulted a significant increase in the grain aspect ratio due to the uncomplete recrystallization process. In terms of the LAGBs distribution, the GB maps of 1P condition revealed a significant increase in the LAGBs’ fraction (Fig. 2d). A significant increase in the LAGBs density of 225% after processing via 1P was depicted compared to the AA sample (Fig. 2c). Accordingly, the UFG structure resulted from ECAP processing through 1P led to increase the fraction of LAGBs which agreed with previous study^35,36^. Shana et al.^35^ reported that during the early passes of ECAP a generation and multiplication of dislocation is occur which is followed by entanglement of the dislocation forming the LAGBs and hence, the density of LAGBs was increased after processing through 1P. The accumulation of the plastic strain up to 4Bc revealed an almost UFG, which indicated that 4Bc led to a complete dynamic recrystallization (DRX) process (Fig. 2e). The grain size was ranged between 0.23 up to 11.7 µm with average grain size of 1.94 µm (the average grain size was decreased by 92.7% compared to the AA condition). On the other hand, 4Bc revealed a decrease in the LAGBs density by 25.4% compared to 1P condition due to the dynamic recovery process. The decrease in the LAGBs density after processing through 4Bc was coupled with an increase in the HAGBs by 4.4% compared to 1P condition (Figs. 2f, 3b). Accordingly, the rise of the HAGBs after multiple passes can be referred to the transfer of LAGBs into HAGBs during the DRX process.

Similar findings were reported in previous studies. Dumitru et al.^36^ reported that ECAP processing resulted in the accumulation and re-arrangement of dislocations which resulted in forming a subgrains and equiaxed grains with an UFG structure and a fully homogenous and equiaxed grain structure for ZK30 alloy was attained after the third pass. Furthermore, they reported that the LAGBs is transferred into HAGBs during the multiple passes which leads to the decrease in the LAGBs density. Figueiredo et al.^37^ reported that the grains evolved during the early passes of ECAP into a bimodal structure while further processing passes resulted in the achievement of a homogenous UFG structure. Zhou et al.^38^ reported that by increasing the processing passes resulted in generation of new grain boundaries which resulted in increasing the misorientation to accommodate the deformation and the Geometrically Necessary Dislocations (GNDs) generated a part of the total dislocations with a HAGBs, thus develop misorientations between the neighbor grains. Tong et al.^39^ reported that the fraction of LAGBs is decreased during multiple passes for Mg–Zn–Ca alloy.

**Figure 4:**
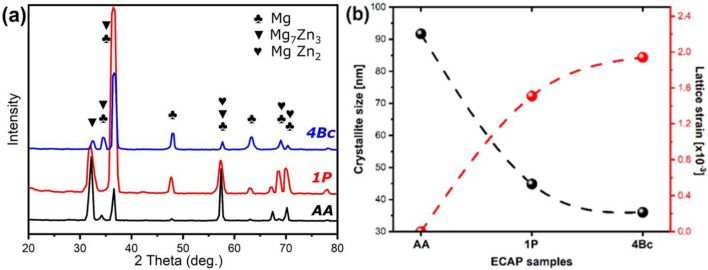
(**a**) XRD patterns for the AA ZK30 alloy and after 1P and 4Bc ECAP processing, (**b**) variations of crystallite size and lattice strain as a function of processing condition using the Williamson–Hall method.

Figure 4a displays X-ray diffraction (XRD) patterns of the AA-ZK30 alloy, 1P, and 4Bc extruded samples, revealing peaks corresponding to primary α-Mg Phase, Mg_7_Zn_3_, and MgZn_2_ phases in all extruded alloys, with an absence of diffraction peaks corresponding to oxide inclusions. Following 1P-ECAP, the α-Mg peak intensity exhibits an initial increase, succeeded by a decrease and fluctuations, signaling texture alterations in the alternative Bc route. The identification of the MgZn_2_ phase is supported by the equilibrium Mg–Zn binary phase diagram^40^. However, the weakened peak intensity detected for the MgZn_2_ phase after the 4Bc–ECAP process indicates that a significant portion of the MgZn_2_ dissolved into the Mg matrix, attributed to their poor thermal stability. Furthermore, the atomic ratio of Mg/Zn for this phase is approximately 2.33, leading to the deduction that the second phase is the Mg_7_Zn_3_ compound. This finding aligns with recent research on Mg–Zn alloys^41^. Additionally, diffraction patterns of ECAP-processed samples exhibit peak broadening and shifting, indicative of microstructural adjustments during plastic deformation. These alterations undergo analysis for crystallite size and micro-strain using the modified Williamson and Hall (W–H) method^42^, as illustrated in Fig. 4b. After a single pass of ECAP, there is a reduction in crystallite size and an escalation in induced micro-strain. Subsequent to four passes-Bc, further reductions in crystallite size and heightened micro-strain (36 nm and 1.94 × 10^–3^, respectively) are observed. Divergent shearing patterns among the four processing routes, stemming from disparities in sample rotation, result in distinct evolutions of subgrain boundaries. Route BC, characterized by the most extensive angular range of slip, generates subgrain bands on two shearing directions, expediting the transition of subgrain boundaries into high-angle grain boundaries^43,44^. Consequently, dislocation density and induced micro-strains reach their top in route BC, potentially influenced by texture modifications linked to orientation differences in processing routes. Hence, as the number of ECAP passes increases, an intensive level of deformation is observed, leading to the existence of dynamic recrystallization and grain refinement, particularly in the ECAP 4-pass. This enhanced deformation effectively impedes grain growth. Consequently, the number of passes in the ECAP process is intricately linked to the equivalent strain, inducing grain boundary pinning, and resulting in the formation of finer grains. The grain refinement process can be conceptualized as a repetitive sequence of dynamic recovery and recrystallization in each pass. In the case of the 4Bc ECAP process, dynamic recrystallization dominates, leading to a highly uniform grain reduction and, causing the grain boundaries to become less distinct^45^. Figure 4b indicates that microstructural features vary with ECAP processing routes, aligning well with grain size and mechanical properties.

#### Wear behavior

**Figure 5:**
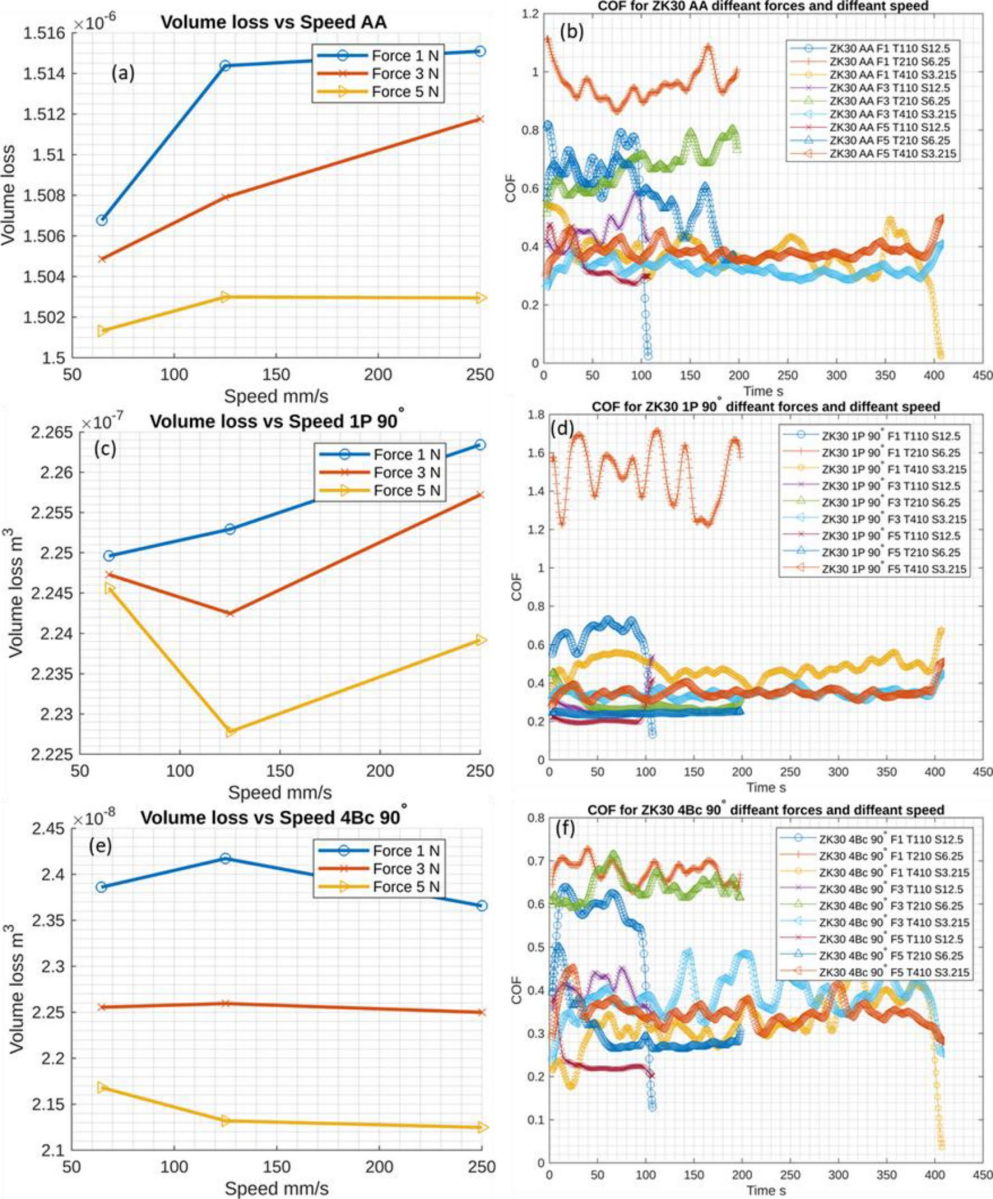
Volume loss of ZK30 alloy (**a**,**c**,**e**) and the average coefficient of friction (**b**,**d**,**f**) in its (**a**,**b**) AA, (**c**,**d**) 1P and (**e**,**f**) 4Bc conditions as a function of different wear parameters.

Figure 5 shows the volume loss (VL) and average coefficient of friction (COF) for the AA and ECAPed ZK30 alloy. The AA billets exhibited the highest VL at all wear parameters compared to the ECAPed billets as shown in Fig. 5. From Fig. 5a it revealed that performing the wear test at applied load of 1N exhibited the higher VL compared to the other applied forces. In addition, increasing the applied force up to 3 N revealed lower VL compared to 1 N counterpart at all wear speeds. Further increase in the applied load up to 5 N revealed a notable decrease in the VL. Similar behavior was attained for the ECAP-processed billets through 1P (Fig. 5c) and 4Bc (Fig. 5e). The VL was improved by increasing the applied load for all samples as shown in Fig. 5 which indicated an enhancement in the wear resistance. Increasing the applied load increases the strain hardening of ZK30 alloy that are in contact as reported by Yasmin et al.^46^ and Kori et al.^47^. Accordingly, increasing the applied load resulted in increasing the friction force, which in turn hinder the dislocation motion and resulted in higher deformation, so that ZK30 experienced strain hardening and hence, the resistance to abrasion is increased, leading to improving the wear resistance^48^. Furthermore, increasing the applied load leads to increase the surface in contact with wear ball and hence, increases gripping action of asperities, which help to reduces the wear rate of ZK30 alloy as reported by Thuong et al.^48^. Out of contrary, increasing the wear speed revealed increasing the VL of the AA billets at all wear loads. For the ECAPed billet processed through 1P, the wear speed of 125 mm/s revealed the lowest VL while the wear speed of 250 mm/s showed the highest VL (Fig. 5c). Similar behaviour was recorded for the 4Bc condition. In addition, from Fig. 5c, it was clear that 1P condition showed higher VL compared to 4Bc (Fig. 5e) at all wear parameters, indicating that processing via multiple passes resulted in significant grain size refinement (Fig. 2). Hence, higher hardness and better wear behavior were attained which agreed with previous study^7^. In addition, from Fig. 5, it was clear that increasing the wear speed increased the VL. For the AA billets tested at 1N load the VL was 1.52 × 10^–6^ m^3^. ECAP processing via 1P significantly improved the wear behavior as the VL was reduced by 85% compared to the AA condition. While compared to the AA condition, the VL improved by 99.8% while straining through 4Bc, which is accounted for by the considerable refinement that 4Bc provides. A similar trend was observed for the ECAPed ZK30 samples tested at a load of 3 and 5 N (Fig. 5). Accordingly, the significant grain refinement after ECAP processing (Fig. 2) increased the grain boundaries area; hence, a thicker oxide protective layer can be formed, leading to improve the wear resistance of the ECAPed samples. It is worth to mentioning here that, the grain refinement coupled with refining the secondary phase particle and redistribution resulted from processing through ECAP processing through multiple passes resulted in improving the hardness, wear behavior and mechanical properties according to Hall–Petch equation^7,13,49^. Similar findings were noted for the ZK30 billets tested at 3 N load, processing through 1P and 4Bc exhibited decreasing the VL by 85%, 99.85%, respectively compared to the AA counterpart. Similar finding was recorded for the findings of ZK30 billets which tested at 5 N load.

From Fig. 5, it can be noticed that the COF curves revealed a notable fluctuation with implementing least square method to smoothing the data, confirming that the friction during the testing of ECAPed ZK30 alloy was not steady for such a time. The remarkable change in the COF can be attributed to the smaller applied load on the surface of the ZK30 samples. Furthermore, the results of Fig. 5 revealed that ECAP processing reduced the COF, and hence, better wear behavior was attained. Furthermore, for all ZK30 samples, it was observed that the highest applied load (5 N) coupled with the lowest wear time (110 s) exhibited better COF and better wear behavior was displayed. These findings agreed with Farhat et al.^50^, they reported that decreasing the grain size led to improve the COF and hence improve the wear behavior. Furthermore, they reported that a plastic deformation occurs due to the friction between contacted surface which resisted by the grain boundaries and fine secondary phases. In addition, the strain hardening resulted from ECAP processing leads to decrease the COF and improving the VL^50^. Sankuru et al.^43^ reported that ECAP processing foe pure Mg resulted in substantial grain refinement which was reflected in improving both microhardness and wear rate of the ECAPed billets. Furthermore, they found that increasing the number of passes up to 4Bc reduced the wear rate by 50% compared to the AA condition. Based on the applied load and wear velocity and distance, wear mechanism can be classified into mild wear and severe wear regimes^49^. Wear test parameters in the present study (load up to 5 N and speed up to 250 mm/s) falls in the mild wear regime where the delamination wear and oxidation wear mechanisms would predominantly take place^43,51^.

**Figure 6:**
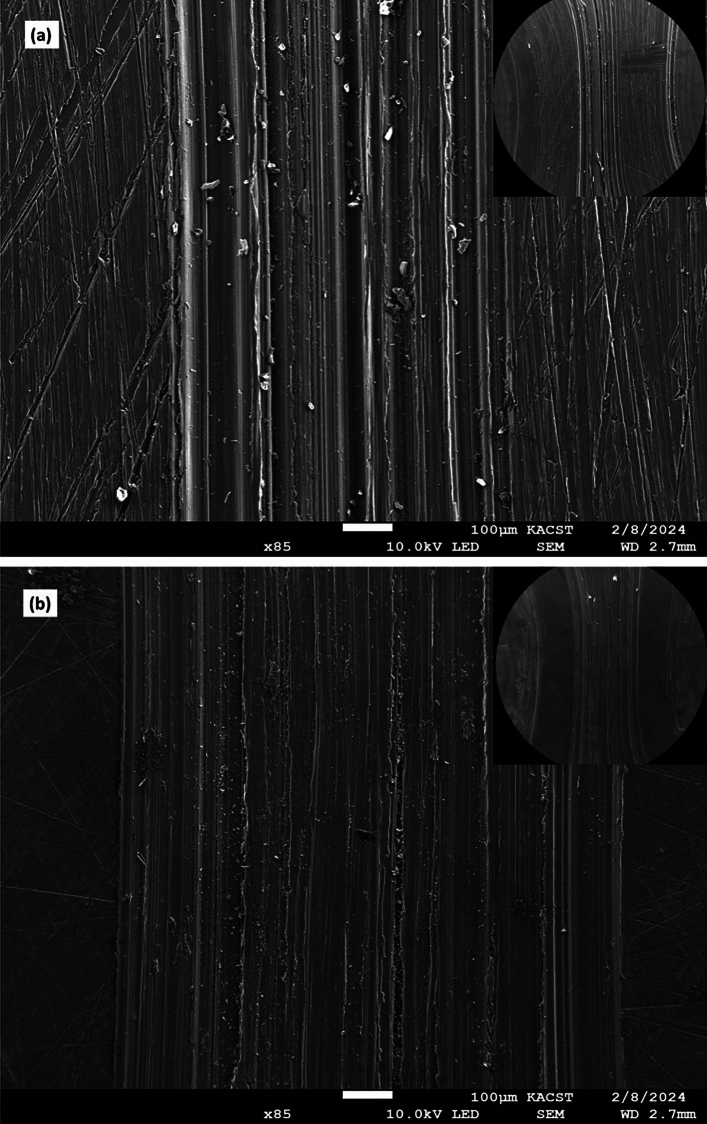
SEM micrograph of the worn surface after the wear test: (**a**–**c**) AA alloy; (**b**) ECAP-processed through 4Bc.

The worn surface morphologies of the ZK30-AA billet and ECAPed billet processed through 4Bc are shown in Fig. 6. From Fig. 6 it can revealed that scores of wear grooves which aligned parallel to the wear direction have been degenerated on the worn surface in both AA (Fig. 6a) and 4Bc (Fig. 6b) conditions. Accordingly, the worn surface was included a combination of adhesion regions and a plastic deformation bands along the wear direction. Furthermore, it can be observed that the wear debris were adhered to the ZK30 worn surface which indicated that the abrasion wear mechanism had occur^52^. Lim et al.^53^ reported that hard particle between contacting surfaces scratches samples and resulted in removing small fragments and hence, wear process was occurred. In addition, from Fig. 6a,b it can depicted that the wear grooves on the AA billet were much wider than the counterpart of the 4Bc sample and which confirmed the effectiveness of ECAP processing in improving the wear behavior of the ZK30 alloy. Based on the aforementioned findings it can be concluded that ECAP-processed billets exhibited enhanced wear behavior which can be attributed to the obtained UFG structure^52^.

### Prediction of wear behavior

#### Regression modeling

Several regression transformations approach and associations among variables that are independent have been investigated in order to model the wear output responses. The association between the supplied parameters and the resulting responses was modeled using quadratic regression. The models created in the course of the experiment are considered statistically significant and can be used to forecast the response parameters in relation to the input control parameters when the highest possible coefficient of regression of prediction (R^2^) is closer to 1. The regression Eqs. (9)–(14) represent the predicted non-linear model of volume loss (VL) and coefficient and friction (COF) at different passes as a function of velocity (V) and applied load (P), with their associated determination and adjusted coefficients. The current study’s adjusted R^2^ and correlation coefficient R^2^ values fluctuated between 95.67 and 99.97%, which is extremely near to unity.

$${\text{AA }}\left\{ {\begin{array}{*{20}l} {VL = + 1.52067 \times 10^{ - 6} - 1.89340 \times 10^{ - 9} P - 4.81212 \times 10^{ - 11} V + 8.37361 \times 10^{ - 12} P * V} \hfill & {} \hfill \\ { - 2.91667E - 10 {\text{P}}^{2} - 2.39989E - 14 {\text{V}}^{2} } \hfill & {(9)} \hfill \\ {\frac{1}{{{\text{COF}}}} = + 2.72098 + 0.278289P - 0.029873V - 0.000208 P \times V + 0.047980 {\text{P}}^{2} } \hfill & {} \hfill \\ { + 0.000111 {\text{V}}^{2} - 0.000622 {\text{P}}^{2} \times V + 6.39031 \times 10^{ - 6} P \times {\text{V}}^{2} } \hfill & {(10)} \hfill \\ \end{array} } \right.$$

$$1{\text{ Pass }}\left\{ {\begin{array}{*{20}l} {VL = + 2.27635 \times 10^{ - 7} + 7.22884 \times 10^{ - 10} P - 2.46145 \times 10^{ - 11} V - 1.03868 \times 10^{ - 11} P \times V} \hfill & {} \hfill \\ { - 1.82621 \times 10^{ - 10} {\text{P}}^{2} + 6.10694 \times 10^{ - 14} {\text{V}}^{2} } \hfill & {} \hfill \\ { + 8.76819 \times 10^{ - 13} P^{2} \times V + 2.48691 \times 10^{ - 14} P \times V^{2} } \hfill & {(11)} \hfill \\ {\frac{1}{{{\text{COF}}}} = - 0.383965 + 1.53600P + 0.013973V - 0.002899 P \times V} \hfill & {} \hfill \\ { - 0.104246 P^{2} - 0.000028 V^{2} } \hfill & {(12)} \hfill \\ \end{array} } \right.$$

$$4{\text{ Pass}}\left\{ {\begin{array}{*{20}l} {VL = + 2.29909 \times 10^{ - 8} - 2.29012 \times 10^{ - 10} P + 2.46146 \times 10^{ - 11} V - 6.98269 \times 10^{ - 12} P \times V } \hfill & {} \hfill \\ { - 1.98249 \times 10^{ - 11} {\text{P}}^{2} - 7.08320 \times 10^{ - 14} {\text{V}}^{2} } \hfill & {} \hfill \\ { + 3.23037 \times 10^{ - 13} P^{2} * V + 1.70252 \times 10^{ - 14} P \times V^{2} } \hfill & {(13)} \hfill \\ {\frac{1}{{{\text{COF}}}} = + 2.77408 - 0.010065P - 0.020097V - 0.003659 P \times V} \hfill & {} \hfill \\ { + 0.146561 P^{2} + 0.000099 V^{2} } \hfill & {(14)} \hfill \\ \end{array} } \right.$$

**Figure 7:**
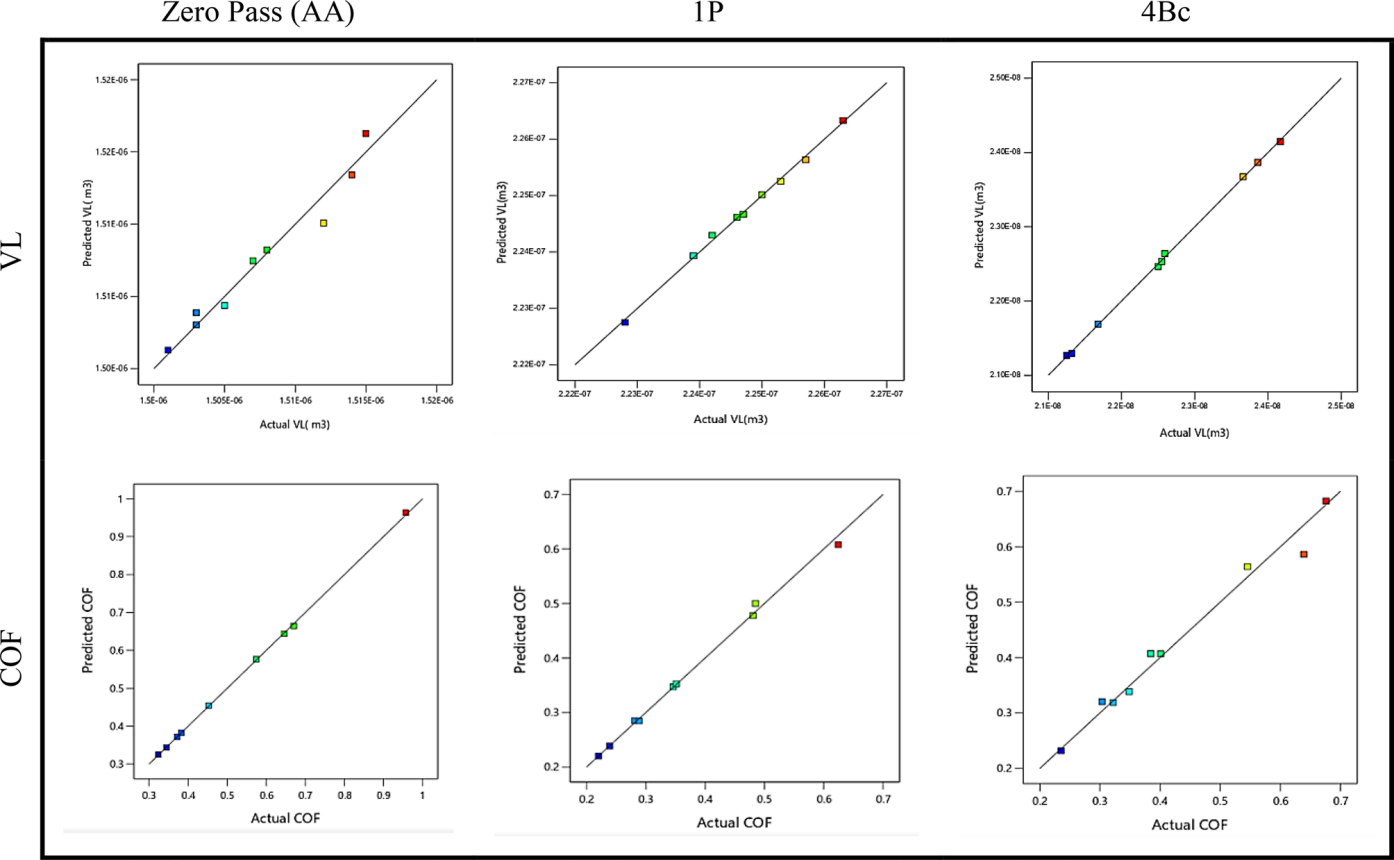
Comparison between VL and COF of experimental and predicted values of ZK30 at AA, 1P, and 4Bc.

The experimental data are plotted in Fig. 7 as a function of the corresponding predicted values for VL and COF for zero pass, one pass, and four passes. The minimal output value is indicated by blue dots, which gradually change to the maximum output value indicated by red points. The effectiveness of the produced regression models was supported by the analysis of these maps, which showed that the practical and projected values matched remarkably well and that the majority of their intersection locations were rather close to the median line.

**Figure 8:**
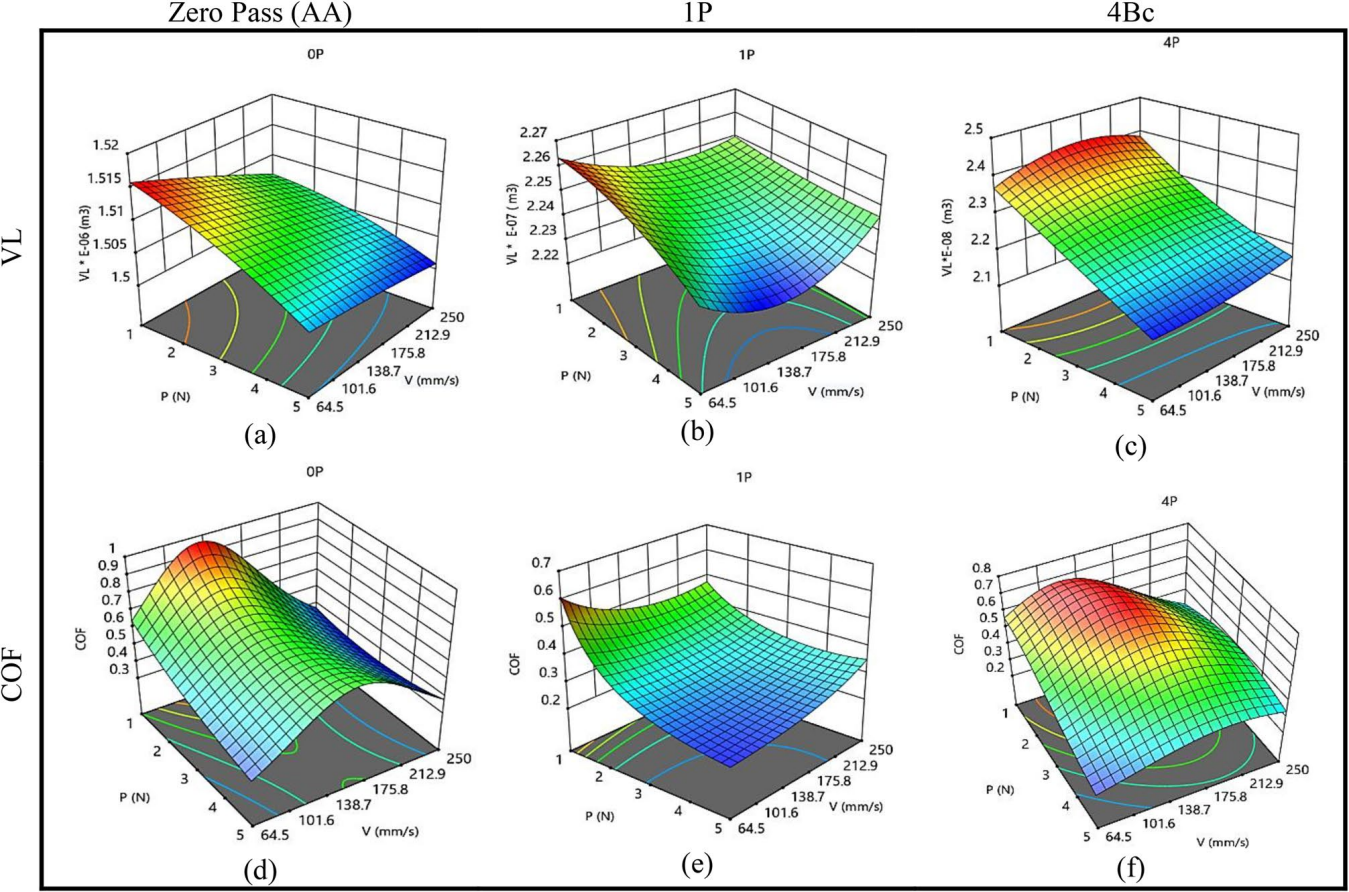
Three-dimensional plot of VL (**a**–**c**) and COF (**d**–**f**) of ZK30 at AA, 1P, and 4Bc.

As a consequence of wear characteristics (P and V), Fig. 8 displays 3D response plots created using regression models to assess changes in VL and COF at various ECAP passes. For VL, the volume loss and applied load exhibit an inverse proportionality at various ECAP passes, which is apparent in Fig. 8a–c. It was observed that increasing the applied load in the wear process will minimize VL. So, the optimal amount of VL was obtained at an applied load of 5N. There is an inverse relation between V of the wear process and VL at different ECAP passes. There is a clear need to change wear speeds for bullets with varying numbers of passes. As a result, the increased number of passes will need a lower wear speed to minimize VL. The minimal VL at zero pass is 1.50085E−06 m^3^ obtained at 5N and 250 mm/s. Also, at a single pass, the optimal VL is 2.2266028E−07 m^3^ obtained at 5 N and 148 mm/s. Finally, the minimum VL at four passes is 2.07783E−08 m^3^ at 5N and 64.5 mm/s.

Figure 8d–f presents the effect of wear parameters P and V on the COF for ECAPed ZK30 billets at zero, one, and four passes. There is an inverse proportionate between the applied load in the wear process and the coefficient of friction. As a result, the minimum optimum value of COF of the ZK30 billet at different process passes was obtained at 5 N. On the other hand, the speed used in the wear process decreased with the number of billet passes. The wear test rates for billets at zero, one, and four passes are 250, 64.5, and 64.5 mm/s, respectively. The minimum COF at zero pass is 0.380134639, obtained at 5N and 250 mm/s. At 5N and 64.5 mm/s, the lowest COF at one pass is 0.220277466. Finally, the minimum COF at four passes is 0.23130154 at 5N and 64.5 mm/s.

#### Machine learning prediction models

The previously mentioned modern ML algorithms have been used here to provide a solid foundation for analyzing the obtained data and gaining significant insights. The following section will give the results acquired by employing these approaches and thoroughly discuss the findings.

**Figure 9:**
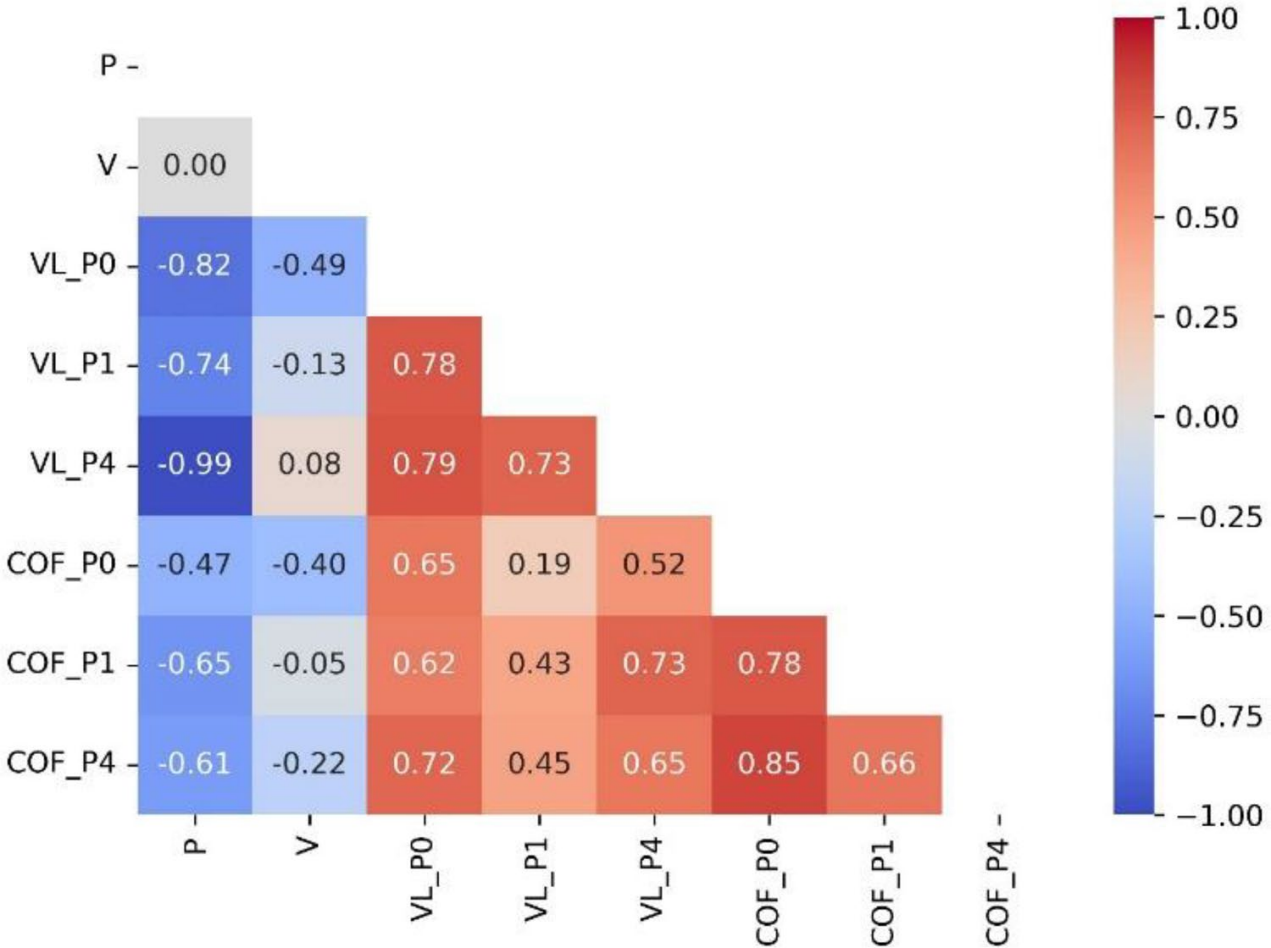
Correlation plots of input and output variables showcasing the strength and direction of relationships between each input–output variable using correlation coefficients.

The correlation plots and correlation coefficients (Fig. 9) between the input variables, force, and speed, and the six output variables (VL_P0, VL_P1, VL_P4, COF_P0, COF_P1, and COF_P4) for data preprocessing of ML models give valuable insights into the interactions between these variables. Correlation charts help to investigate the strength and direction of a linear relationship between model input and output variables. We can initially observe if there is a positive, negative, or no correlation between each two variables by inspecting the scatterplots. This knowledge aids in comprehending how changes in one variable effect changes in the other. In contrast, the correlation coefficient offers a numerical assessment of the strength and direction of the linear relationship. It ranges from − 1 to 1, with near − 1 indicating a strong negative correlation, close to 1 indicating a strong positive correlation, and close to 0 indicating no or weak association. It is critical to examine the size and importance of the correlation coefficients when examining the correlation between the force and speed input variables and the six output variables (VL_P0, VL_P1, VL_P4, COF_P0, COF_P1, and COF_P4). A high positive correlation coefficient implies that a rise in one variable is connected with an increase in the other. In contrast, a high negative correlation coefficient indicates that an increase in one variable is associated with an increase in the other. From Fig. 9 it was clear that for all ZK30 billets, the both VL and COP were reversely proportional with the applied (in the range of 1-up to- 5N). Regarding the wear speed, the VL of both the AA and 1P conditions exhibited an inversed proportional with the wear speed while 4Bc exhibited a direct proportional with the wear speed (in the range of 64.5- up to- 250 mm/s) despite of the COP for all samples revealed an inversed proportional with the wear speed. The VL of AA condition (P0) revealed strong negative correlation coefficient of − 0.82 with the applied load while it displayed intermediate negative coefficient of − 0.49 with the wear speed. For 1P condition, VL showed a strong negative correlation of − 0.74 with the applied load whereas it showed a very weak negative correlation coefficient of − 0.13 with the speed. Furthermore, the VL of 4Bc condition displayed a strong negative correlation of − 0.99 with the applied load while it displayed a wear positive correlation coefficient of 0.08 with the speed. Similar trend was observed for the COF, the AA, 1P and 4Bc samples displayed intermediate negative coefficient of − 0.047, − 0.65 and − 0.61, respectively with the applied load while it showed a weak negative coefficient of − 0.4, − 0.05 and − 0.22, respectively with wear speed.

**Figure 10:**
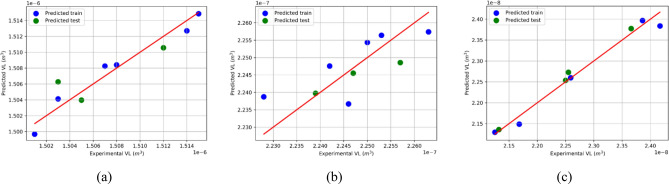
Predicted train and predicted test VL versus actual data computed for different applied loads and number of passes of (**a**) 0P (AA), (**b**) 1P, and (**c**) 4Bc: evaluating the performance of the VL prediction best model achieved using LR algorithm.

Figure 10 shows the predicted train and test VL values compared to the original data, indicating that the VL prediction model performed well utilizing the LR (Linear Regression) technique. The R^2^-score is a popular statistic for assessing the goodness of fit of a regression model. It runs from 0 to 1, with higher values indicating better performance. In this scenario, the R^2^-scores for both the training and test datasets range from 0.55 to 0.99, indicating that the ML model has established a significant correlation between the projected VL values and the actual data. This shows that the model can account for a considerable percentage of the variability in VL values.

**Figure 11:**
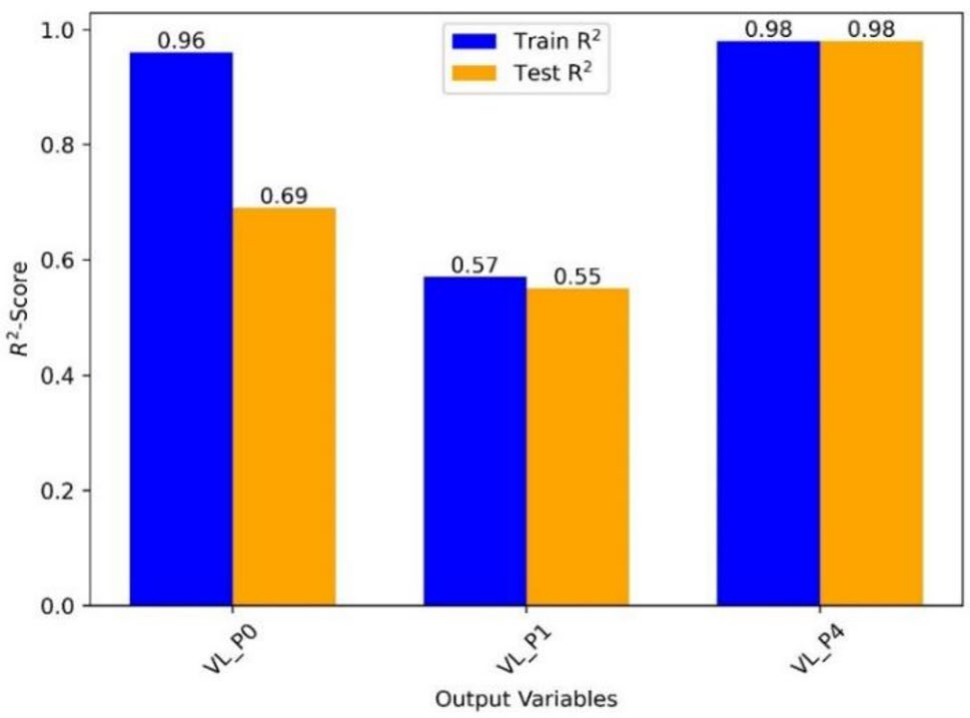
Result summary of ML train and test sets displaying R^2^-score for each model.

The R^2^-scores for training and testing three distinct ML models for the output variables ‘VL_P0’, ‘VL_P1’, and ‘VL_P4’ are summarized in Fig. 11. The R^2^-score, also known as the coefficient of determination, is a number ranging from 0 to 1 that indicates how well the model fits the data. For VL_P0, R^2^ for testing is 0.69, and that for training is 0.96, indicating that the ML model predicts the VL_P0 variable with reasonable accuracy on unknown data. On the other hand, the R^2^ value of 0.96 for training suggests that the model fits the training data rather well. In summary, the performance of the ML models changes depending on the output variables. With R^2^ values of 0.98 for both training and testing, the model predicts 'VL_P4' with great accuracy. However, the model’s performance for 'VL_P0' is reasonable, with an R^2^ score of 0.69 for testing and a high R^2^ score of 0.96 for training. The model’s performance for 'VL_P1' is relatively poor, with R^2^ values of 0.55 for testing and 0.57 for training. Additional assessment measures must be considered to understand the models' prediction capabilities well. Therefore, as presented in the following section, we did no-linear polynomial fitting with extracted equations that accurately link the output and input variables.

**Figure 12:**
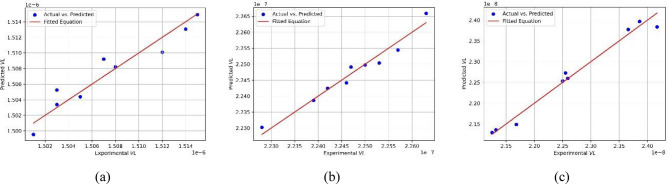
Predicted versus actual (**a**) VL_P0 fitted to Eq. 15 with R^2^-score of 0.92, (**b**) VL_P1 fitted to Eq. 16 with R^2^-score of 0.96, (**c**) VL_P4 fitted to Eq. 17 with R^2^-score of 0.98.

Furthermore, the data was subjected to polynomial fitting with first- and second-degree models (Fig. 12). The fitting accuracy of the data was assessed using the R^2^-score, which ranged from 0.92 to 0.98, indicating a good fit. The following equations (Eqs. 15 to 17) were extracted from fitting the experimental dataset of the volume loss at different conditions of applied load (P) and the speed (V) as follows:

15 $${\text{VL}}\_{\text{P}}0 = 1.519e - 06{ } + { } - 2.417e - 09{\text{ * P }} + { } - 3.077e - 11{ * }V$$

16 $$VL\_{\text{P}}1 = 2.299e - 07 - 5.446e - 10 * {\text{P}} - 5.431e - 11 * V - 5.417e - 11 * {\text{P}}^{2} + 2.921e - 12 * {\text{P}} V + 1.357e - 13 * V^{2}$$

17 $$VL\_{\text{P}}4 = 2.433e - 08 - 6.200e - 10 * {\text{P}} + 1.042e - 12 * V$$

**Figure 13:**
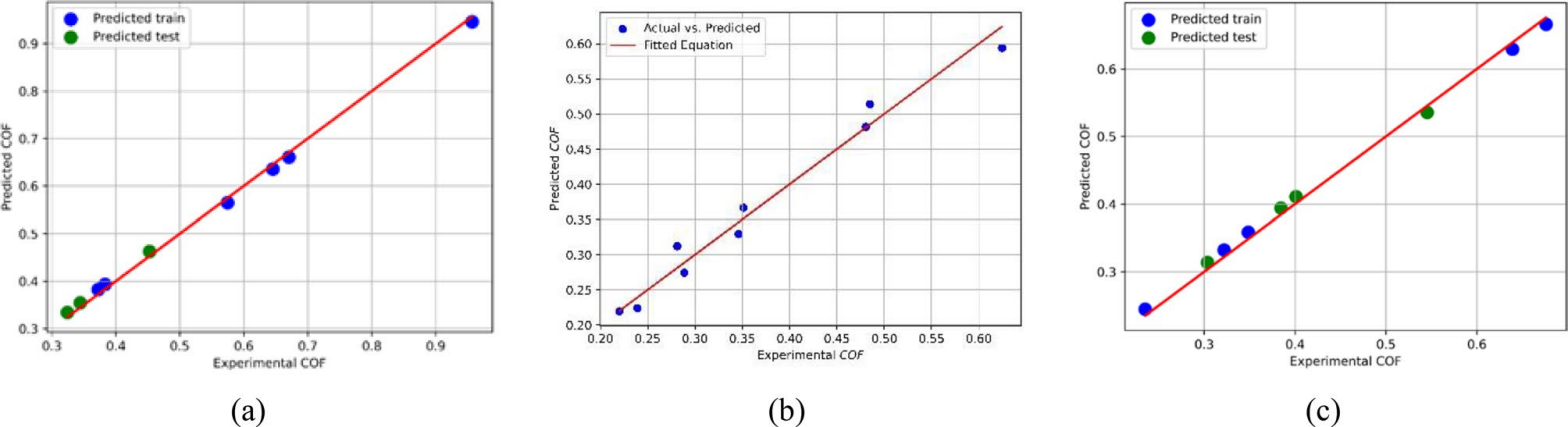
Predicted train and predicted test COF versus actual data computed for different applied loads and number of passes of (**a**) 0P (AA), (**b**) 1P, and (**c**) 4Bc: evaluating the performance of the COF prediction best model achieved using SVM and GPR algorithms.

Figure 13 depicts the predicted train and test coefficients of friction (COF) values placed against the actual data. The figure seeks to assess the performance of the best models obtained using the SVM (Support Vector Machine) and GPR (Gaussian Process Regression) algorithms for various applied loads and number of passes (0, 1P, and 4P). The figure assesses the accuracy and efficacy of the COF prediction models by showing the predicted train and test COF values alongside the actual data. By comparing projected and actual data points, we may see how closely the models match the true values. The ML models trained and evaluated on the output variables 'COF_P0', 'COF_P1', and 'COF_P4' using SVM and GPR algorithms show great accuracy and performance, as summarized in Fig. 13. The R^2^ ratings for testing vary from 0.97 to 0.99, showing that the models efficiently capture the predicted variables' variability efficiently. Furthermore, the training R^2^ scores are consistently high at 0.99, demonstrating a solid fit to the training data. These findings imply that the ML models can accurately predict the values of 'COF_P0', 'COF_P1', and 'COF_P4' and generalize well to new unseen data.

**Figure 14:**
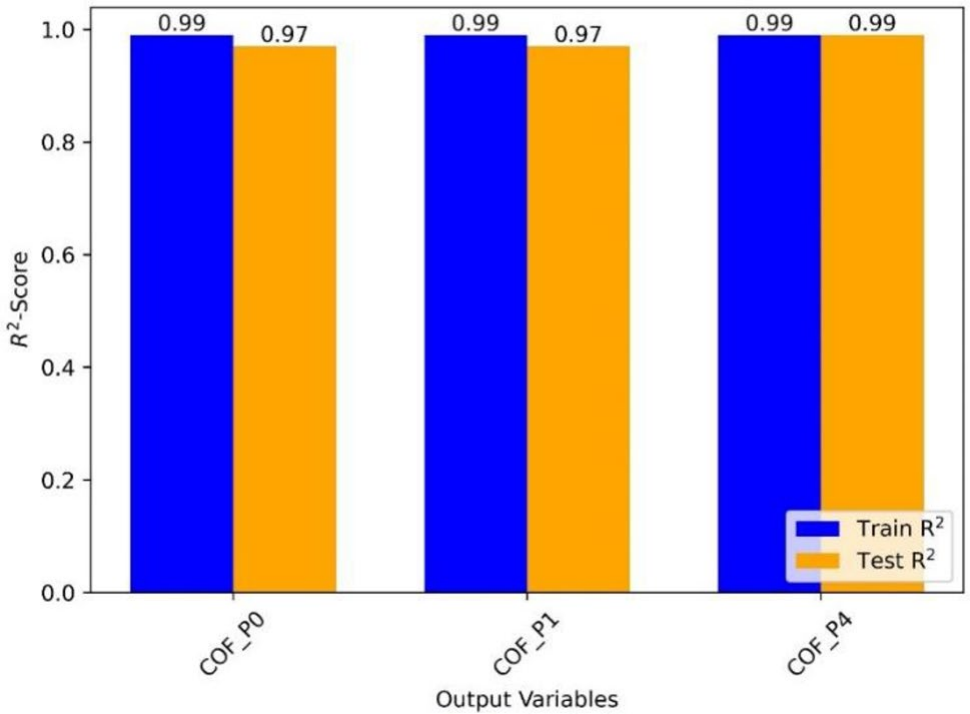
Result summary of ML train and test sets displaying R^2^-score for each model.

Figure 14 presents a summary of the results obtained through machine learning modeling. The R^2^ values achieved for COF modeling using SVM and GPR are 0.99 for the training set and range from 0.97 to 0.99 for the testing dataset. These values indicate that the models have successfully captured and accurately represented the trends in the dataset.

### Optimization of wear behavior

#### Optimization by response surface methodology (RSM)

The results of the RSM optimization carried out on the volume loss and coefficient of friction at zero pass (AA), along with the relevant variables, are shown in Appendix A-1. The red and blue dots represented the wear circumstance (P and V) and responses (VL and COF) for each of the ensuing optimization findings. The volume loss and coefficient of friction optimization objective were formed to “in range,” using “minimize” as the solution target, and the expected result of the desirability function was in the format of “smaller-is-better” attributes. The values of (A) P = 5 N and (B) V = 250 mm/s were the optimal conditions for volume loss. Appendix A-1(a) shows that this resulted in the lowest volume loss value attainable of 1.50127E-6 m^3^. Also, the optimal friction coefficient conditions were (A) P = 2.911 N and (B) V = 250 mm/s. This led to the lowest coefficient of friction value possible, which was 0.324575, as shown in Appendix A-1(b).

Appendix A-2 displays the outcomes of the RSM optimization performed on the volume loss and coefficient of friction at one pass, together with the appropriate variables. The volume loss and coefficient of friction optimization objectives were designed to be "in range," with "minimize" as the solution objective. It was anticipated that the intended function would provide "smaller-is-better" traits. The ideal conditions for volume loss were (A) P = 4.95 N and (B) V = 136.381 mm/s. This yielded the lowest volume loss value feasible of 2.22725E-7 m^3^, as seen in Appendix A-2 (a). The optimal P and V values for the coefficient of friction were found to be (A) P = 5 N and (B) V = 64.5 mm/s. As demonstrated in Appendix A-2 (b), this resulted in the lowest coefficient of friction value achievable, which was 0.220198.

Similarly, Appendix A-3 displays the outcomes of the RSM optimization performed on the volume loss and coefficient of friction at four passes, together with the appropriate variables. The volume loss and coefficient of friction optimization objectives were designed to be "in range," with "minimize" as the solution objective. The desired function’s expected result would provide of "smaller-is-better" characteristics. The optimal conditions for volume loss were (A) P = 5 N and (B) V = 77.6915 mm/s. This yielded the lowest volume loss value feasible of 2.12638E-8 m^3^, as seen in Appendix A-1 (a). The optimal P and V values for the coefficient of friction were found to be (A) P = 4.95612 N and (B) V = 64.9861 mm/s. As seen in Appendix A-1(b), this resulted in the lowest coefficient of friction value achievable, which was 0.235109.

#### Optimization by genetic algorithm and hybrid DOE-GA

The most appropriate combination of wear-independent factors that contribute to the minimal feasible volume loss and coefficient of friction was determined using a genetic algorithm (GA). Based on genetic algorithm technique, the goal function for each response was determined by taking Eqs. (9)–(14) and subjecting them to the wear boundary conditions, P and V. The following expression applies to the recommended functions for objective: Minimize (VL, COF), subjected to ranges of wear conditions: 1 ≤ P ≤ 5 (N), 64.5 ≤ V ≤ 250 (mm/s).

**Figure 15:**
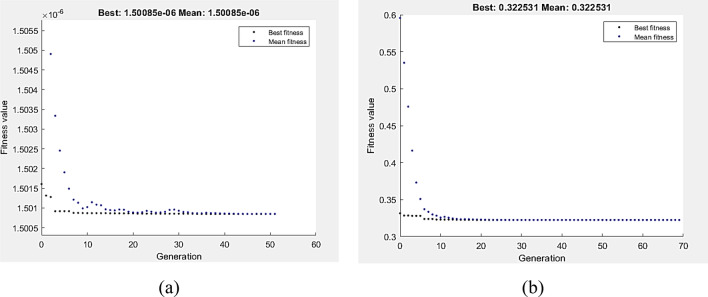
Optimum VL (**a**) and COF (**b**) by GA at AA condition.

**Figure 16:**
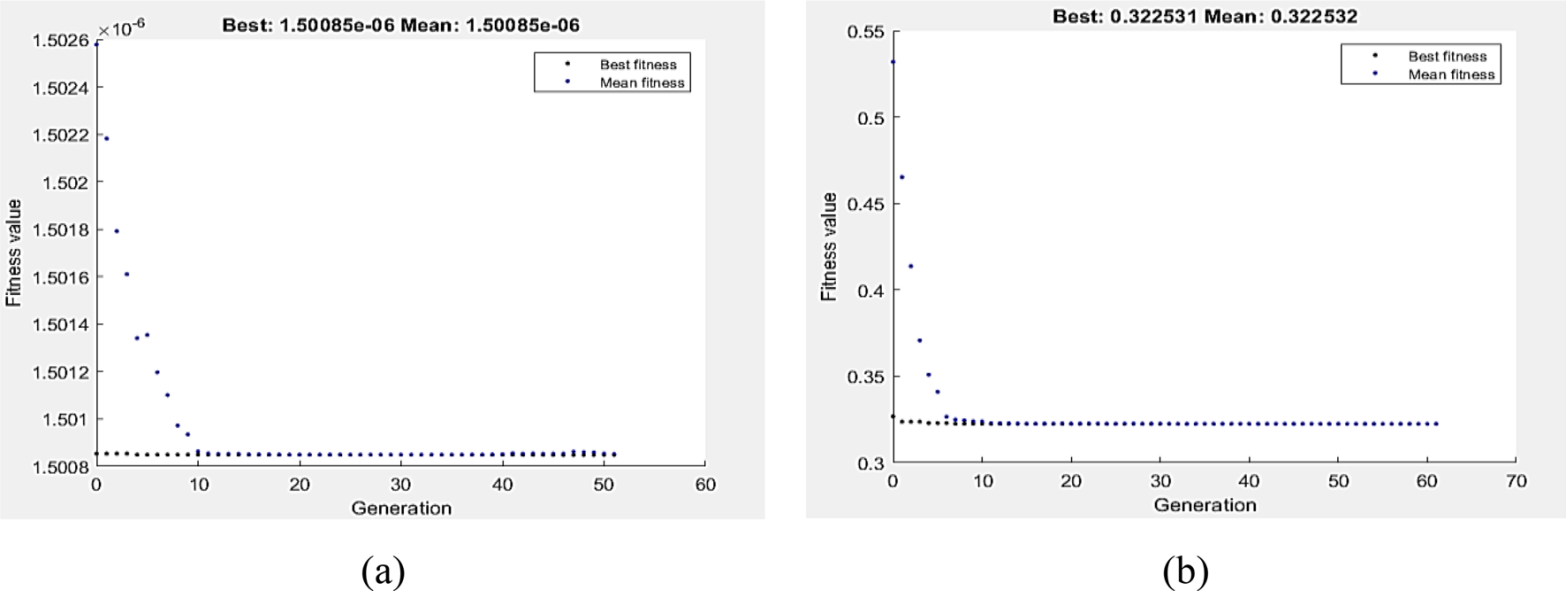
Optimum VL (**a**) and COF (**b**) by hybrid DOE-GA at AA condition.

Figures 15 and 16 show the GA optimization technique’s performance in terms of fitness value and the running solver view, which were derived from MATLAB, together with the related wear requirements for the lowest VL and COF at zero pass. VL and COF were suggested to be minimized by Eqs. (9) and (10), which were then used as the function of fitness and exposed to the wear boundary limit. According to Fig. 15a, the lowest value of VL that GA could find was 1.50085E−6 m^3^ at P = 5N and V = 249.993 mm/s. Furthermore, the GA yielded a minimum COF value of 0.322531 at P = 2.91 N and V = 250 mm/s (Fig. 15b).

The DOE–GA hybrid analysis was carried out to enhance the GA outcomes. Wear optimal conditions of VL and COF at zero pass are used to determine the initial populations of hybrid DOE–GA. The hybrid DOE–GA yielded a minimum VL value of 1.50085E-6 m^3^ at a speed of 249.993 mm/s and a load of 5N (Fig. 16a). Similarly, at a 2.91 N and 250 mm/s speed load, the hybrid DOE–GA yielded a minimum COF (Fig. 16b) of 0.322531.

**Figure 17:**
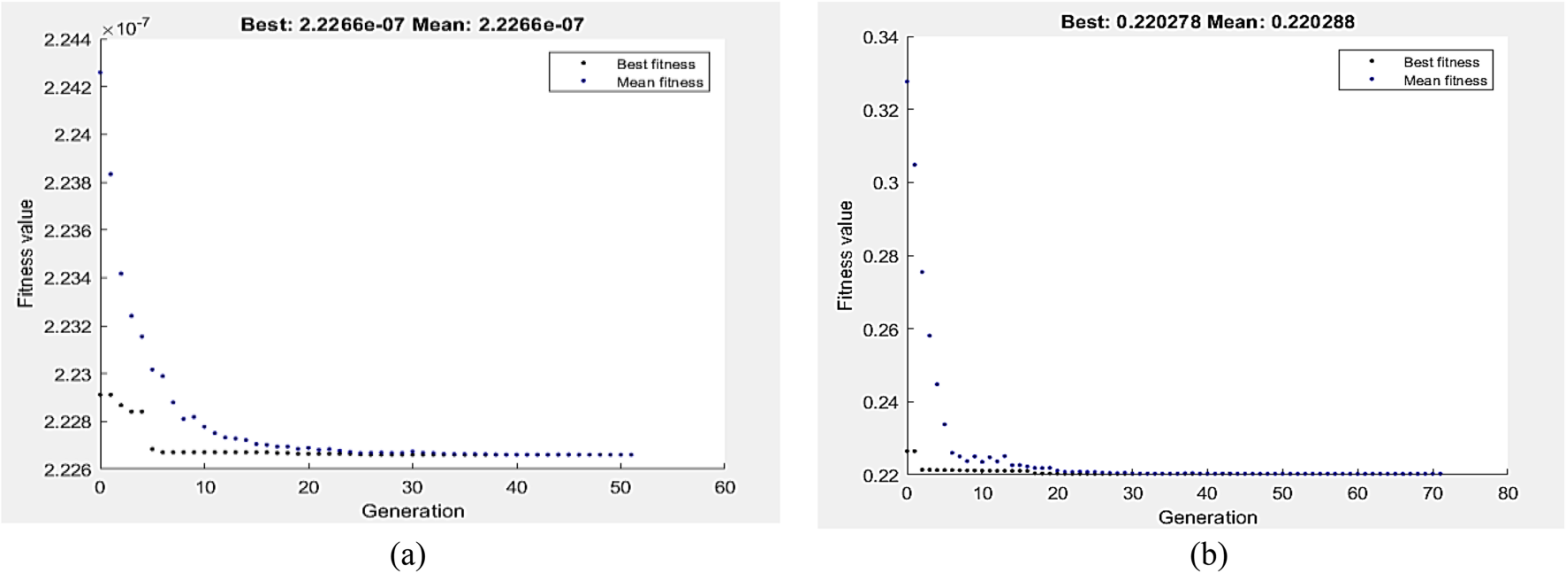
Optimum VL (**a**) and COF (**b**) by GA at 1P condition.

**Figure 18:**
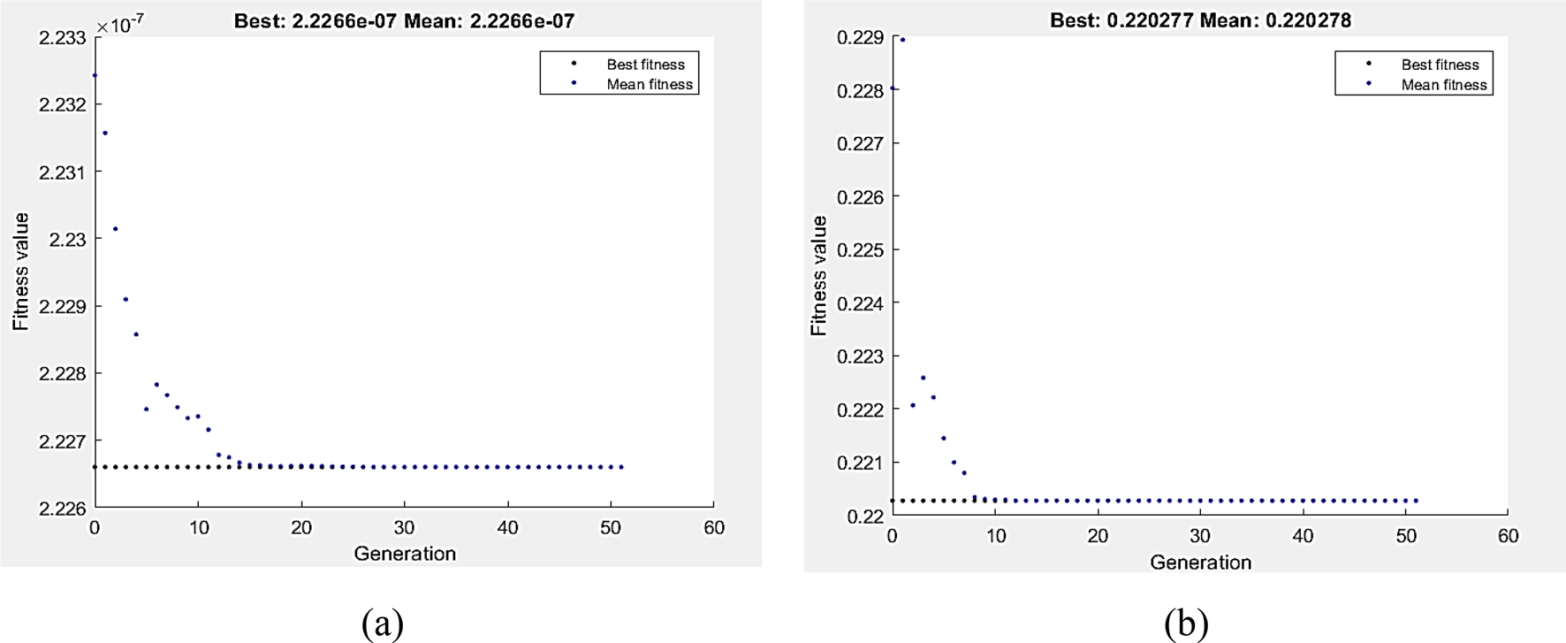
Optimum VL (**a**) and COF (**b**) by hybrid DOE-GA at 1P condition.

The fitness function, as defined by Eqs. 11 and 12, was the depreciation of VL and COF at a 1P, subject to the wear boundary condition. Figure 17a,b display the optimal values of VL and COF by GA, which were 2.2266E−7 m^3^ and 0.220278, respectively. The lowest VL measured at 147.313 mm/s and 5 N. In comparison, 5 N and 64.5 mm/s were the optimum wear conditions of COF as determined by GA. Hybrid DOE–GA results of minimum VL and COF at a single pass were 2.2266 E-7 m^3^ and 0.220278, respectively, obtained at 147.313 mm/s and 5 N for VL as shown in Fig. 18a and 5 N and 64.5 mm/s for COF as shown in Fig. 18b.

**Figure 19:**
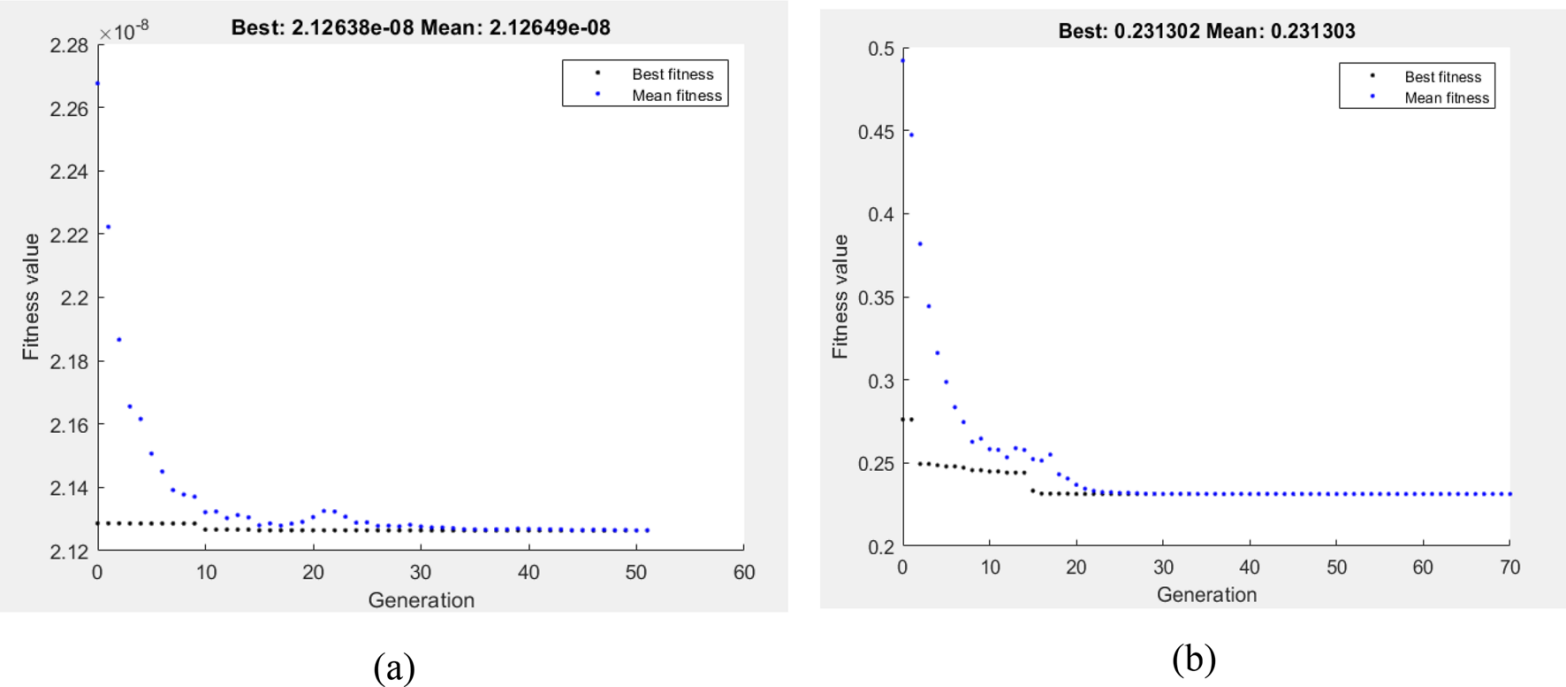
Optimum VL (**a**) and COF (**b**) by GA at 4Bc condition.

**Figure 20:**
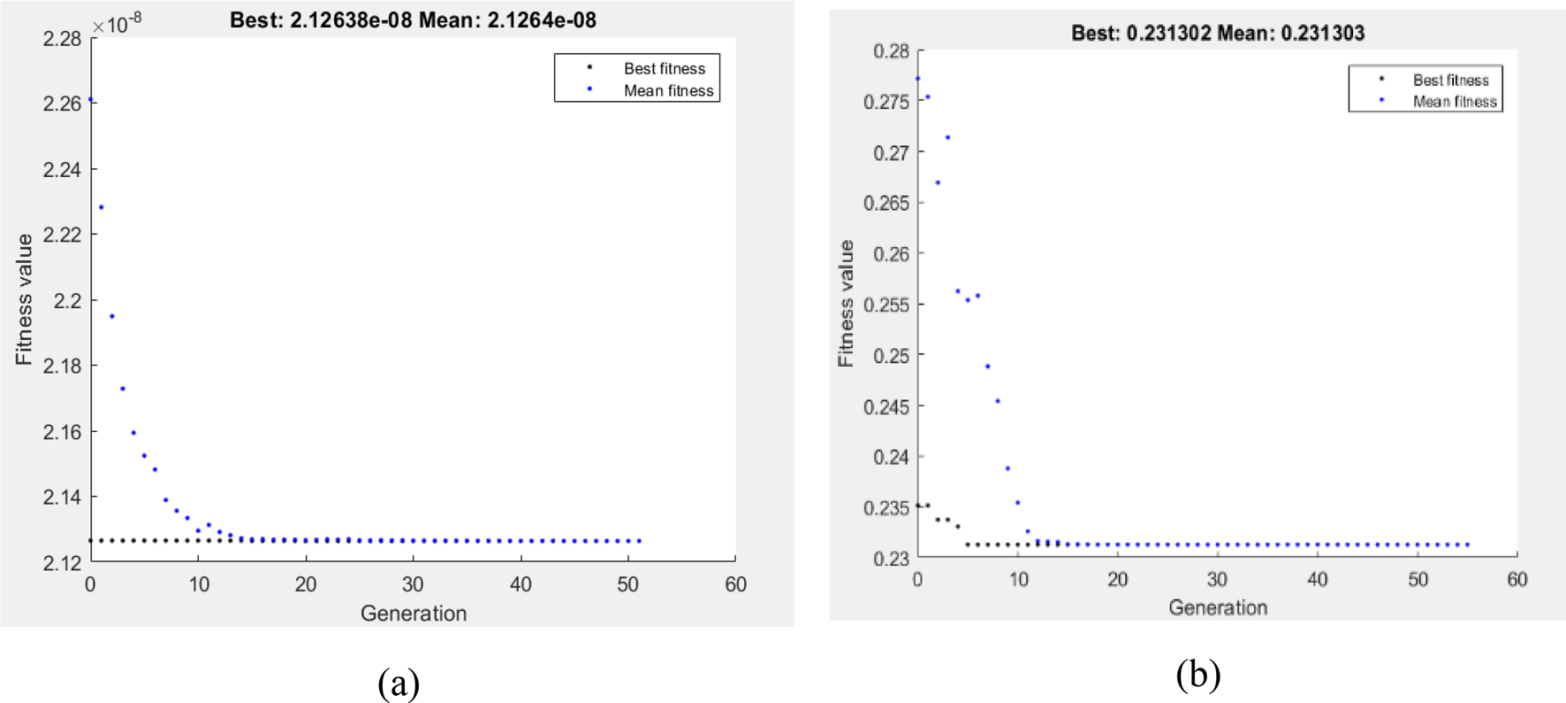
Optimum VL (**a**) and COF (**b**) by hybrid DOE-GA at 4Bc condition.

Subject to the wear boundary condition, the fitness function was the minimization of VL and COF at four passes, as defined by Eqs. 13 and 14. The optimum values of VL and COF via GA shown in Fig. 19a,b were 2.12638E−8 m^3^ and 0.231302, respectively. The lowest reported VL was 5 N and 77.762 mm/s. However, GA found that the optimal wear conditions for COF were 5 N and 64.5 mm/s. In Fig. 20a,b, the hybrid DOE–GA findings for the minimum VL and COF at four passes were 2.12638E−8 m^3^ and 0.231302, respectively. These results were achieved at 77.762 mm/s and 5 N for VL and 5 N and 64.5 mm/s for COF.

#### Optimization by multi-objective genetic algorithm (MOGA)

A mathematical model whose input process parameters influence the quality of the output replies was solved using the multi-objective genetic algorithm (MOGA) technique^54^. In the current study, the multi-objective optimization using genetic algorithm (MOGA) as the objective function, regression models, was implemented using the GA Toolbox in MATLAB 2020 and the P and V are input wear parameter values served as the top and lower bounds, and the number of parameters was set to three. After that, the following MOGA parameters were selected: There were fifty individuals in the initial population, 300 generations in the generation, 20 migration intervals, 0.2 migration fractions, and 0.35 Pareto fractions. Constraint-dependent mutation and intermediary crossover with a coefficient of chance of 0.8 were used for optimization. The Pareto optimum, also known as a non-dominated solution, is the outcome of MOGA. It is a group of solutions that consider all of the objectives without sacrificing any of them^55^.

By addressing both as multi-objective functions was utilized to identify the lowest possible values of the volume loss and coefficient of friction at zero pass. Equations (9) and (10) were the fitness functions for volume loss and coefficient of friction at zero pass for ZK30. The Pareto front values for the volume loss and coefficient of friction at zero pass, as determined by MOGA, are listed in Table 2. The volume loss (Objective 1) and coefficient of friction (Objective 2) Pareto chart points at zero pass are shown in Fig. 21. A friction coefficient reduction due to excessive volume loss was observed. As a result, giving up a decrease in the coefficient of friction can increase volume loss. For zero pass, the best volume loss was 1.50096E−06 m^3^ with a sacrifice coefficient of friction of 0.402941. However, the worst volume loss was 1.50541E−06 m^3^, with the best coefficient of friction being 0.341073.

**Table 2:**
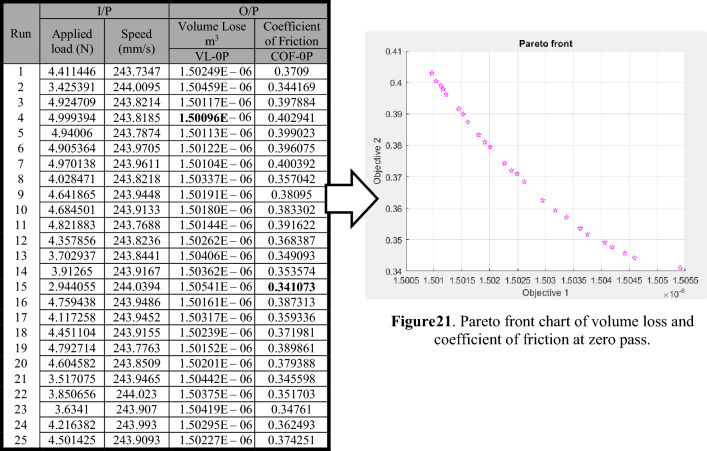
Paterian points of volume loss and coefficient of friction at zero pass.

The genetic algorithm was used for the multi-objective functions of minimal volume loss and coefficient of friction. The fitness functions for volume loss and coefficient of friction at one pass were represented by Eqs. (11) and (12), respectively. Table 3 displays the Pareto front points of volume loss and coefficient of friction at one pass. Figure 22 presents the volume loss (Objective 1) and coefficient of friction (Objective 2) Pareto chart points for a single pass. It was discovered that the coefficient of friction decreases as the volume loss increases. As a result, the volume loss can be reduced at the expense of a higher coefficient of friction. The best volume loss for a single pass was 2.22699E−07 m^3^, with the worst maximum coefficient of friction being 0.242371 and the best minimum coefficient of friction being 0.224776 at a volume loss of 2.23405E−07 m^3^.

**Table 3:**
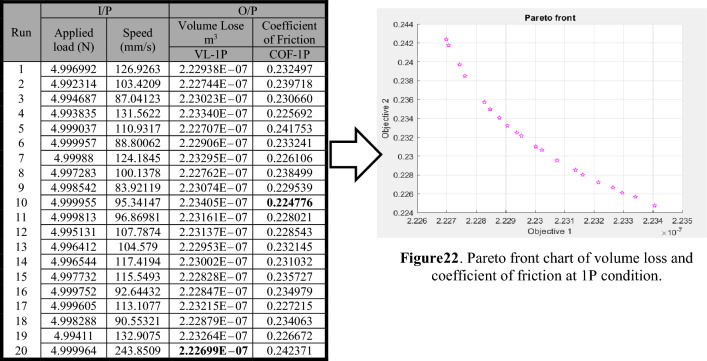
Paterian points of volume loss and coefficient of friction at 1P condition.

The multi-objective functions of minimal volume loss and coefficient of friction were handled by Eqs. (13) and (14), respectively, served as the fitness functions for volume loss and coefficient of friction at four passes. The Pareto front points of volume loss and coefficient of friction at four passes are shown in Table 4. The Pareto chart points for the volume loss (Objective 1) and coefficient of friction (Objective 2) for four passes are shown in Fig. 23. It was shown that when the volume loss increases, the coefficient of friction lowers. The volume loss can be decreased as a result, however, at the expense of an increased coefficient of friction. The best minimum coefficient of friction was 0.2313046 at a volume loss of 2.12663E−08 m^3^, and the best minimum volume loss was 2.126397E−08 m^3^ at a coefficient of friction of 0.245145 for four passes. In addition, Table 5 compares wear response values at DOE, RSM, GA, hybrid RSM-GA, and MOGA.

**Table 4:**
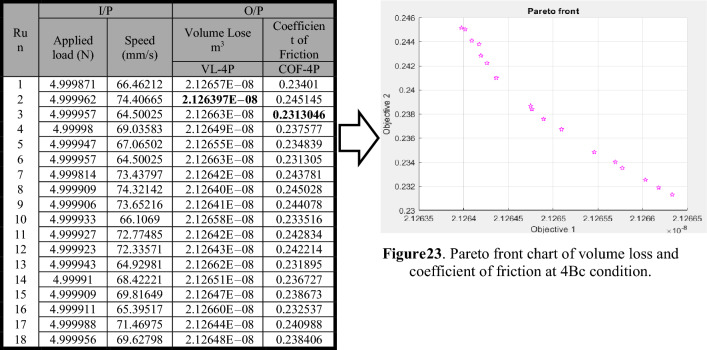
Paterian points of volume loss and coefficient of friction at 4Bc condition.

**Table 5:** Summary results of ZK30 wear process.

No. of pass	Response		DOE	RSM	GA	DOE-GA	MOGA	
							VL	COF
AA	VL	Value	1.501E−6	1.50127E−6	1.50085E−6	1.50085E−6	1.50096E−06	
		Load	5	5	5	5	4.999394	
		Speed	250	250	249.993	249.997	243.8185	
	COF	Value	0.3242	0.324575	0.322531	0.322531		0.341073
		Load	3	2.911	2.91	2.91		2.944055
		Speed	250	250	250	250		244.0394
1P	VL	Value	2.228E−7	2.22725E−7	2.2266E−7	2.2266E−7	2.22699E−07	
		Load	5	4.95	5	5	4.999964	
		Speed	125	136.381	147.313	147.313	243.8509	
	COF	Value	0.2201	0.220198	0.220278	0.220277		0.224776
		Load	5	5	5	5		4.999955
		Speed	64.5	64.5	64.5	64.5		95.34147
4Bc	VL	Value	2.125E−8	2.12638E−8	2.12638E−8	2.12638E−8	2.126397E−08	
		Load	5	5	5	5	4.999962	
		Speed	64.5	77.6915	77.762	77.762	74.40665	
	COF	Value	0.2351	0.235109	0.231302	0.231302		0.2313046
		Load	5	4.95612	5	5		4.999957
		Speed	64.5	64.9861	64.5	64.5		64.50025

#### Optimization of large space

This section proposed the optimal wear parameters of different responses, namely VL and COF of ZK30. The presented optimal wear parameters, such as P and V, are based on previous studies of ZK30 that recommended the applied load from one to 30 N and speed from 64.5 to 1000 mm/s. Table 6 presents the optimal condition of the wear process of different responses by genetic algorithm (GA).

**Table 6:** Validated wear response based on previous studies.

No. of pass		AA	1P	4Bc
VL	Value	1.25E−06	1.18E−06	6.94E−09
	Load	30	30	30
	Speed	263.4	64.5	64.5
COF	Value	0.00980	0.209	0.0079
	Load	5.627	6.47	30
	Speed	1000	64.5	64.5

### Validation of RSM and ML models for ZK30 processing through ECAP

#### Validation of RSM models

Table 7 displays the validity of wear’s regression model for VL under several circumstances. The wear models' validation was achieved under various load and speed conditions. The volume loss response models had the lowest error % between the practical and regression models and were the most accurate, based on the validation data. Table 7 indicates that the data unambiguously shows that the predictive molding performance has been validated, as shown by the reasonably high accuracy obtained, ranging from 69.7 to 99.9%.

**Table 7:** Regression models and experimental data for VL response validation.

No. of pass	Condition		VL			
	P	V	Experimental	Regression	Error %	Accuracy
AA	4	100	2.14625565388639E−06	1.5067300E−06	29.79727288	70.203
	4	300	2.15350616550351E−06	1.5018818E−06	30.25876484	69.741
	10	125	2.13238193912871E−06	1.4766462E−06	30.75132777	69.249
	10	175	2.13401493214397E−06	1.4780669E−06	30.73774138	69.262
1P	4	100	2.13183672084049E−07	2.2399680E−07	5.072211305	94.928
	4	300	2.11721747110203E−07	2.2641394E−07	6.939389217	93.061
	10	125	2.03714954514774E−07	2.1634167E−07	6.198227698	93.802
	10	175	2.13274522681493E−07	2.1894805E−07	2.660197991	97.340
4Bc	4	100	2.19359795943822E−08	2.1915585E−08	0.092974167	99.907
	4	300	2.22212270661075E−08	2.2067575E−08	0.691464363	99.309
	10	125	1.95542678089314E−08	1.8658153E−08	4.582709604	95.417
	10	175	1.89954730607354E−08	1.9504023E−08	2.677213869	97.323

#### Validation of ML models

Equations (15 to 17) provide insights into the relationship that links the volume loss with applied load and speed, allowing us to understand how changes in these factors affect the volume loss in the given system. The validity of this modeling was further examined using a new unseen dataset by which the prediction error and accuracy were calculated, as shown in Table 8. Table 8 shows that the data clearly demonstrates that the predictive molding performance has been validated, as evidenced by the obtained accuracy ranging from 69.7 to 99.9%, which is reasonably high.

**Table 8:** Validation results of VL modeling using new unseen dataset.

No. of pass	Condition		VL			
	P	V	Experimental	Regression	Error % (%)	Accuracy (%)
AA	4	100	2.14625565E−06	1.50625500E−06	29.8	70.2
	4	300	2.15350617E−06	1.50010100E−06	30.3	69.7
	10	125	2.13238194E−06	1.49098375E−06	30.1	69.9
	10	175	2.13401493E−06	1.48944525E−06	30.2	69.8
1P	4	100	2.13183672E−07	2.23949280E−07	− 5.0	95.0
	4	300	2.11721747E−07	2.26280080E−07	− 6.9	93.1
	10	125	2.03714955E−07	2.18019812E−07	− 7.0	93.0
	10	175	2.13274523E−07	2.18800312E−07	− 2.6	97.4
4Bc	4	100	2.19359796E−08	2.19542000E−08	− 0.1	99.9
	4	300	2.22212271E−08	2.21626000E−08	0.3	99.7
	10	125	1.95542678E−08	1.82602500E−08	6.6	93.4
	10	175	1.89954731E−08	1.83123500E−08	3.6	96.4

## Conclusions

This research presents a comparative study of the wear behavior of ECAPed ZK30 alloys using experimental, statistical, and machine learning techniques. Different ECAP processing conditions have been implemented, including the processing routes and number of pressing passes. The wear behavior of ECAPed ZK30 alloy has been thoroughly examined in terms of volume loss and coefficient of friction under applied loads and speeds. Prediction and optimization of the wear test parameters of the ECAPed ZK30 samples have been performed via different statistical and machine learning approaches. Finally, another set of experimental conditions has validated models obtained from RSM and ML. The following conclusions could be drawn:

1. ECAP process leads to significant grain refinement, particularly with 4Bc, results in the formation of fine grains. The average grain size of ECAPed ZK30 has significantly decreased by 92.7% compared to the AA condition, reaching an average size of 1.94 µm.
2. ECAP processing, through 1P and 4Bc routes, demonstrates a substantial enhancement in wear resistance. The wear volume loss (VL) has shown remarkable reductions of 85% and 99.8%, respectively, compared to the AA condition.
3. The fluctuation in coefficient of friction (COF) curves during testing of ECAPed ZK30 alloy, attributed to smaller applied loads, indicates non-steady friction behavior. However, overall, ECAP results in a reduction in COF, signifying improved wear behavior.
4. The regression models of VL and COF have correlation coefficient R^2^, and adjusted R^2^ values in the present research ranged from 95.67 to 99.97%, indicating that the experimental and predicted values agreed exceptionally well.
5. The 3D plots reveal that the minimal VL at different ECAP passes was obtained at the highest condition of the wear test.
6. The minimal COF for all ECAP passes was obtained at maximum wear load. However, the optimal speed in the wear process decreased with the number of passes.
7. ML prediction model has established a significant correlation between the projected and the actual data with R^2^-score ranging from 0.92 to 0.98 for VL and from 0.97 to 0.99 for COF.
8. There is good overlap between the wear response values of the DOE-obtained experimental findings and the optimization results from RSM, GA, MOGA, and hybrid DOE–GA.
9. The validation of predicted ML models and VL regression under different wear conditions have an accuracy range of 70% to 99.7%.

## Supplementary Information

Supplementary Information.

## Data Availability

Data is provided within the manuscript and the supplementary information files.
