# Supplementary material for: Optimization of wear parameters for ECAP-processed ZK30 alloy using response surface and machine learning approaches: a comparative study
Source: Sci Rep. 2024 Apr 22;14:9233. doi: 10.1038/s41598-024-59880-0 (PMC11576740; doi:10.1038/s41598-024-59880-0)
Supplement: Supplementary file 1 — Supplementary Information. [file 41598_2024_59880_MOESM1_ESM.pdf]

Appendices

Appendix A

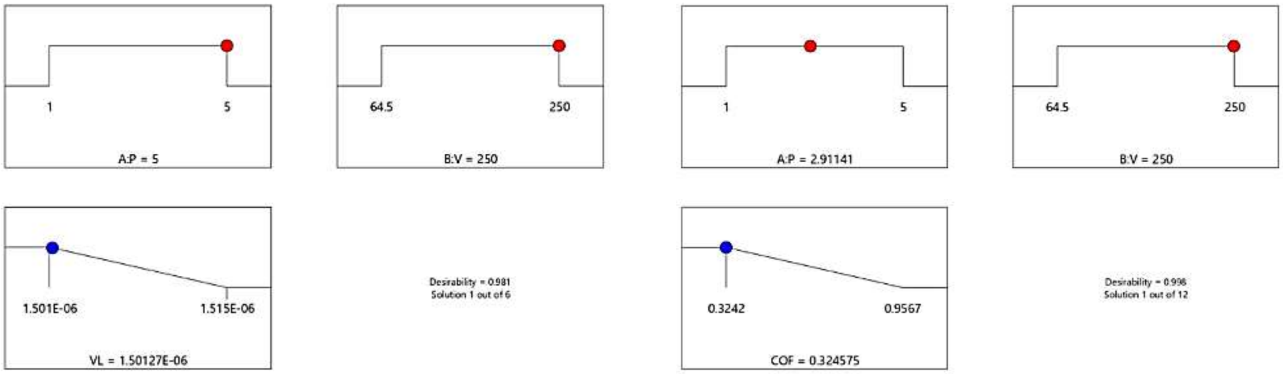

(a) (b)

A-1: RSM optimization results of (a) VL and (b) COF of ZK30 at AA condition

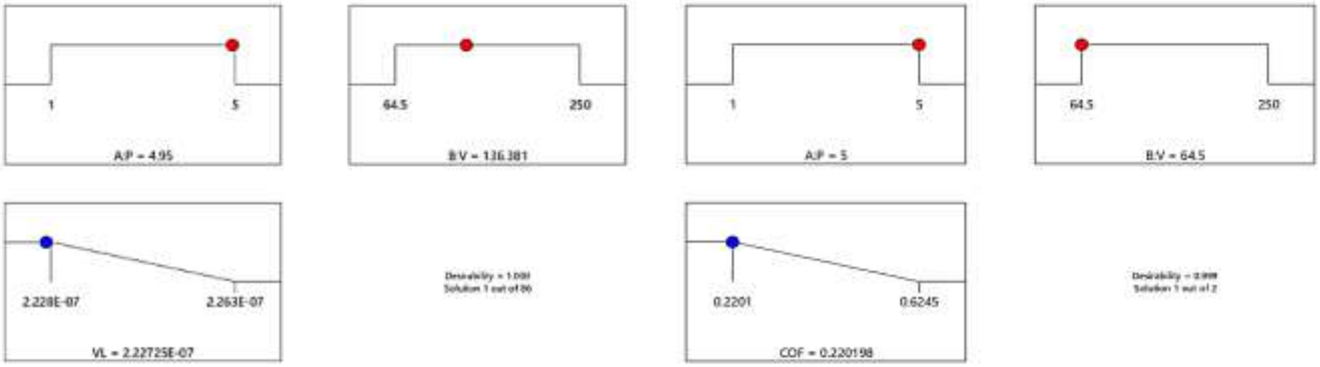

(a) (b)

A-2: RSM optimization results of (a) VL and (b) COF of ZK30 at 1P condition

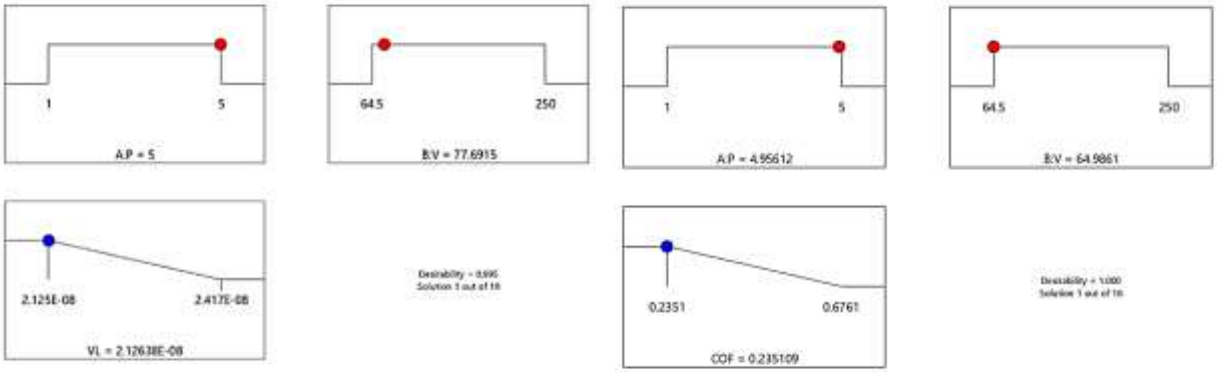

(a) (b)

A-3: RSM optimization results of (a) VL and (b) COF of ZK30 at 4Bc condition
